# Uncovering Trophic Interactions in Arthropod Predators through DNA Shotgun-Sequencing of Gut Contents

**DOI:** 10.1371/journal.pone.0161841

**Published:** 2016-09-13

**Authors:** Débora P. Paula, Benjamin Linard, Alex Crampton-Platt, Amrita Srivathsan, Martijn J. T. N. Timmermans, Edison R. Sujii, Carmen S. S. Pires, Lucas M. Souza, David A. Andow, Alfried P. Vogler

**Affiliations:** 1 Embrapa Genetic Resources and Biotechnology, Parque Estação Biológica, Brasília, Brazil; 2 Department of Life Sciences, Natural History Museum, Cromwell Rd, London, United Kingdom; 3 Department of Genetics, Evolution and Environment, Faculty of Life Sciences, University College London, Gower Street, London, United Kingdom; 4 Department of Biological Sciences, National University of Singapore, Singapore, Singapore; 5 Department of Life Sciences, Imperial College London, Silwood Park Campus, Ascot, United Kingdom; 6 Department of Natural Sciences, Hendon Campus, Middlesex University, The Burroughs, London, United Kingdom; 7 Department of Entomology, University of Minnesota, St. Paul, Minnesota, United States of America; Tierarztliche Hochschule Hannover, GERMANY

## Abstract

Characterizing trophic networks is fundamental to many questions in ecology, but this typically requires painstaking efforts, especially to identify the diet of small generalist predators. Several attempts have been devoted to develop suitable molecular tools to determine predatory trophic interactions through gut content analysis, and the challenge has been to achieve simultaneously high taxonomic breadth and resolution. General and practical methods are still needed, preferably independent of PCR amplification of barcodes, to recover a broader range of interactions. Here we applied shotgun-sequencing of the DNA from arthropod predator gut contents, extracted from four common coccinellid and dermapteran predators co-occurring in an agroecosystem in Brazil. By matching unassembled reads against six DNA reference databases obtained from public databases and newly assembled mitogenomes, and filtering for high overlap length and identity, we identified prey and other foreign DNA in the predator guts. Good taxonomic breadth and resolution was achieved (93% of prey identified to species or genus), but with low recovery of matching reads. Two to nine trophic interactions were found for these predators, some of which were only inferred by the presence of parasitoids and components of the microbiome known to be associated with aphid prey. Intraguild predation was also found, including among closely related ladybird species. Uncertainty arises from the lack of comprehensive reference databases and reliance on low numbers of matching reads accentuating the risk of false positives. We discuss caveats and some future prospects that could improve the use of direct DNA shotgun-sequencing to characterize arthropod trophic networks.

## Introduction

Understanding the complexity of trophic interactions and their causes and consequences has been a major focus of ecology since Elton [1] developed the concept, continuing to the present with about 250 scientific papers each year. However, as many trophic interactions are infrequent and hard to see [2], especially for small arthropods, important trophic links are under-represented. Molecular analyses of predator gut contents have become the dominant approach to identify arthropod trophic interactions [3–6], especially PCR-based methods.

Lately, several studies have combined classic single DNA barcoding with next-generation sequencing, referred to as metabarcoding, which has overcome the limitations of Sanger sequencing when working with mixtures of templates, as those present in the gut [7,8]. However, these PCR-based methods have various limitations that restrict their general applicability and overall scope. Specifically, standard barcode markers (*e*.*g*., *cox1*, rRNAs) are not sufficiently conserved across taxonomic groups to develop universal or generic primers without target match problems [9–11]. Thus, taxonomic biases and inaccurate estimation of the relative abundance of some taxa are common due to marker choice and variation in primer efficiency [11–18].

In addition, in most cases, the feeding by an arthropod on other arthropods limits the use of universal barcode primers. More generally, the selection of primers made by the researcher determines the target organisms and target genes, which constrains the research questions that can be addressed from the outset, e.g. targeting either eukaryotic or bacterial genomes, but not both. This constrains the characterization of complex food webs of generalist predators, as not all prey species can be anticipated and trophic links are often unknown. PCR-based approaches are particularly problematic for the analysis of intraguild predation [19] among closely related species and require the development of stringently diagnostic primers for each species. In these cases of taxonomic proximity between the target prey taxa and the focal predator, sequences of the latter prevail in PCR due to their much higher abundance and lower degradation compared to ingested DNA, unless ‘blocking’ primers can be designed that discriminate efficiently against the host [7,20] or PCR products are sequenced deeply. Together, these various constraints limit the scope of metabarcoding, which performs best on target groups in a narrow taxonomic window within which primer efficiency is relatively uniform, and at the same time the target groups are taxonomically distant from the host organism (e.g. plant chloroplast DNA in the gut of insects; e.g. [21]). Even where these conditions are fulfilled, the PCR-based analysis of ingested (degraded) DNA requires the use of short fragments, which can result in poor taxonomic resolution [22]. Whereas several attempts have been made to circumvent the limitations due to short fragment length (*e*.*g*., [23–25]), biases associated with PCR still remain [11,18,26].

A more suitable approach to construct complex trophic interaction networks and to study multiple interactions at various trophic levels is needed that combines high taxonomic breadth and resolution [11,27]. Recently, metagenomics pipelines have been developed for studying the arthropod diversity in a specimen mixture [16,28,29]. However, in gut content analysis an additional challenge for using metagenomics is posed by the target environmental sample, which is digested and degraded DNA, precluding the use of existing metagenomic pipelines (assembly > binning > annotation of the metadata [30]). Using feeding trials, Srivathsan *et al*. [22] and Paula *et al*. [31] identified diet composition using an alternative metagenomic shotgun-sequencing pipeline of matching unassembled reads from faeces or gut contents with reference databases, and filtering for high overlap length and identity. The sensitivity and specificity of this method depends on the DNA reference databases. As there is no limitation to the identification of taxa imposed by specific molecular markers, the outcome of this approach promises a broader identification of foreign species, including prey, internal parasites and associated microbiomes.

In this work, we aimed to test the power of this alternative DNA shotgun-sequencing pipeline to construct a qualitative trophic interaction network of generalist arthropod predators that are considered to be effective biocontrol agents. These include three species of ladybirds (Coleoptera: Coccinellidae) and an earwig (Dermaptera: Forficulidae), which feed on various herbivorous insects, in particular aphids and moths, and potentially also on the juvenile stages of each other. We used the Illumina platform for shotgun-sequencing of DNA isolated from gut contents of field-collected specimens and an unfed predator as a control. In parallel, we constructed DNA reference databases through mining of publicly available sequence information, including partial and complete mitochondrial and nuclear genomes, and barcode sequences, complemented with sequences of taxa expected to interact with these predators in the sampled habitat. We were able to identify the taxonomic composition of foreign DNA in the guts of these predators. This includes those species directly preyed upon, but also produced a broader picture of the associated organisms, such as parasitoids and microbiomes, for the identification of the trophic interaction links with good taxonomic breadth and resolution. However, the lack of complete reference databases limits the ability to identify all prey taxa, and the low recovery of matched reads limits the sensitivity of this approach and accentuates the need to reduce the risk of false detection of spurious reads possibly generated from sample contamination or highly degraded DNA. This initiative is an effort to develop a satisfactory methodology to determine the targets of various predators that could be used in biological control.

## Material and Methods

### Insects

The predator coccinellids *Cycloneda sanguinea* (*n* = 5), *Harmonia axyridis* (*n* = 1) and *Hippodamia convergens* (*n* = 6), and the dermapteran *Doru luteipes* (*n* = 10), were collected at two organic farms in central Brazil (15°58'27.67"S, 47°29'49.94"W, and 15°49'28.01"S, 48°15'9.66"W) during November 2012. The farms produced similar crops in small fields, including cabbage, cassava, lettuce, and tomato, surrounded by leucaena, banana, coffee and timber trees. All specimens were immediately immersed in 100% ethanol (species were kept separate) and stored at -80°C until total DNA extraction. All collections were authorized by SISBIO (authorization number 36950), and access to the genetic heritage and transportation of biological material was authorized by IBAMA (authorization number 02001.008598/2012-42). Field-collected *H*. *axyridis* pupae were allowed to emerge in the laboratory, and unfed adults (<24 h) were stored at -80°C in 100% ethanol, and designated as a control.

### Shotgun-sequencing of the predator gut contents

The methodological pipeline is illustrated in Figure A in S1 File. To prevent cross contamination among samples, all the preparation procedures (i.e. gut dissection, DNA extraction, quantification) for each predator sample were done on separate days with different tools and materials (tweezers, scalpels, and petri dishes), which were washed with neutral soap, soaked in 5% bleach for 30 min and washed in 70% ethanol. Prior to sample preparation, the bench and instruments were cleaned using 70% ethanol. Guts from the same species were pooled (the control was pooled separately) immediately after dissection in the first buffer of the DNA extraction kit using a DNA-free microtube placed in ice. The DNA extraction, estimation of DNA concentration, and sample quality check were done as described in Paula *et al*. [31]. The DNA concentration across samples was normalized to 20 ng/μl and TruSeq libraries were constructed. All predator gut contents were sequenced on an Illumina MiSeq for 250 bp single-end reads (300 cycles, mean insert size 300 bp, v2 chemistry, proportion of the flow cell: 16.1% for *Cy*. *sanguinea*, 19.8% for *D*. *luteipes*, 27.6% for *H*. *axyridis*), except for *Hi*. *convergens* at 10% of a run and 250 bp paired-end reads (500 cycles, insert size 600–900 bp bp, v2 chemistry) and the control *H*. *axyridis* at 17% of a run and 250 bp paired-end reads (500 cycles, insert size 450 bp, v2 chemistry). For these last two samples, library preparations and sequencing were done in a separate flow cell, months apart from that of the other predators, preventing cross-contamination. All the programs and settings used in the bioinformatics analyses were based on Paula *et al*. [31]. The quality assessment for each dataset was done using FastQC v0.10.1 (http://www.bioinformatics.babraham.ac.uk/projects/fastqc/). Library index adapters were trimmed using Trimmomatic v0.30 (ILLUMINACLIP:2:30:10) [32]. The reads were passed through a quality control check using PrinSEQ v0.19.2 [33], with a minimum Phred quality score of 20. The retained reads after quality control were converted to Fasta format using a CAT and Perl script [31].

### DNA reference databases

Six DNA reference databases (Fasta format) with different taxonomic resolution were constructed for DNA identification: a) Insecta mitogenomes, obtained by downloading all available (at November 2013) insect mitogenomes of 588 species from GenBank (Table A in S1 File), supplemented by sequencing the mitogenomes of 17 potential prey species (Tables B and C in S1 File) with long-range PCR or shotgun methodology [31,34,35]; b) *cytochrome oxidase 1* (*cox1*) barcode sequences of 58,367 Insecta species using a MegaBLAST based pipeline implemented by Hunt *et al*. [36] and adapted by Srivathsan *et al*. [22,37]; c) aphid nuclear genomes obtained from AphidBase (http://www.aphidbase.com/) consisting of the preliminary genome assemblies of *Myzus persicae* and *Aphis gossypii*, and the complete nuclear genome of *Acyrthosiphon pisum* (assembly Acyr_2.0; placed and unplaced scaffolds; GenBank Assembly ID: GCA_000142985.2); d) parasitoid barcode sequences (ITS, rRNA genes, elongation factor, and *cytb*) from known parasitoid genera of the predators and aphids available at GenBank; e) rRNA genes containing data on 3,000,000 bacteria, 150,000 archaea, and 250,000 eukaryote sequences obtained from the SILVA rRNA database (release 115) [38]; f) genomes of bacteria genera known to include insect endosymbionts [31]: *Arsenophonus*, *Blattabacterium*, *Buchnera*, *Cardinium*, *Hamiltonella*, *Midichloria*, *Nosema*, *Regiella*, *Rickettsia*, *Rickettsiella*, *Serratia*, *Spiroplasma* and *Wolbachia*.

### Prey and other foreign DNA detection

After quality control, reads from each predator gut content dataset were matched against the DNA reference databases using BLASTn v2.2.27+ (E-value <1e-5; maximum target sequences 3; no dust) (for Insecta mitogenome, parasitoids, and rRNA databases) and MegaBLAST 2.2.27+ (E-value <1e-9) (for *cox1*, aphid genomes and bacterial genome databases) [39]. The matched reads were filtered for minimum overlap length of 225 bp and identity of 95% (for the bacterial genome database), 98% (for the mitogenome database) and 99% (for all other reference databases) using a set of Python customized scripts to eliminate false taxon identifications as in Paula *et al*. [31] and Srivathsan *et al*. [22,37] (https://github.com/asrivathsan/readidentifier). The mapping region for each matched read was checked manually. Reads matching to the following regions were discarded: a) control region of mitogenomes; b) nuclear repeat regions, such as short sequence repeats-SSR; c) Coccinellini rRNA because this region does not differentiate prey from predator. Using these rigorous criteria, a matched read was assigned to a taxon for the mitogenome, *cox1*, aphid genomes and bacterial genome databases. For the rRNA and parasitoid databases, taxon assignment was made to the lowest taxonomic level that could be reliably identified.

## Results

### Prey and other foreign DNA detected in the predator guts

The Illumina sequencing of the libraries of the four predator species generated >2 million reads per species after quality control. Several thousand reads matched across the mitogenome of the respective focal predator (Figures B-E in S1 File) ranging from 0.07% to 0.7% of total reads depending on the library (Table 1). An additional 15 to 239 reads per library were identified as insect mitochondrial DNA but foreign to the focal predator (Table 1). The highest number of foreign insect taxa identified based on these reads was found in *Hi*. *convergens* (n = 8, Table 2). For the other libraries, the numbers ranged from two to three, and were not related to the number of pooled predator specimens. In addition, non-insect DNA reads were found using the various databases (Table 1). *Doru luteipes* had the highest richness of symbionts (nine genera), plants, fungi and non-symbiont bacteria, while *Cy*. *sanguinea* had the lowest. We did not detect foreign reads in the gut of the unfed recently emerged (control) *H*. *axyridis*, except for one widespread and ubiquitous bacterium (Table 1).

**Table 1:** Number of Reads and Taxa (in parentheses) in the Gut Content of the Predators.

Predator	Total reads^a^	Predator	Foreign DNA			
		mtDNA	Insecta	Symbionts	Plant	Fungi
*Cycloneda sanguinea*	2,837,177	2,061	15 (3)	21 (2)	1	1
*Hippodamia convergens*	2,647,833	10,506	239 (8)	102 (3)	1	13 (1)
*Harmonia axyridis*	3,440,064	9,216	180 (4)	309 (7)	2 (2)	3 (1)
*Doru luteipes*	2,183,902	17,428	27 (4)	3,032 (9)	130 (2)	577 (2)
Control-group^b^	3,502,252	7,427	0	0 (0)	0	0

^a^ After trimming index library and quality control.

^b^ Recently emerged *H*. *axyridis* adults without feeding.

The taxonomic assignment of the foreign DNA in the gut content of each predator is presented in Table 2, along with the number of reads matched to each of the DNA reference databases. There were fewer matches and fewer species identified using the *cox1* database than the mitogenome database, consistent with the ~20x greater size of the mitogenome and the greater number of target genes which were hit apparently randomly (Figures B-E in S1 File). In addition, to search for aphid prey we also used available whole-genome sequences to map the shotgun reads, which revealed the presence of *Aphis gossypii* in the *H*. *axyridis* gut, consistent with the matches to the Insecta mitogenomes (Table 2).

**Table 2:** Foreign Taxon and Corresponding Number of Reads (in parentheses) Detected in the Gut Content of each Predator using Different DNA Reference Databases.

Predator	DNA reference databases					
	Insecta mitogenomes	*cox1*	Aphid genomes	Parasitoids	Bacterial genomes	rRNA
*Cycloneda sanguinea*	*Doru luteipes* (3)	*Harmonia axyridis* (4)	-	Chalcidoidea (5)	*Hamiltonella* sp. (20)^a^	Ascomycota (1)
	*Harmonia axyridis* (3)				*Spiroplasma* spp. (1)	Spermatophyta (1)
*Hippodamia convergens*	*Dinocampus coccinellae* (58)	*Dinocampus coccinellae* (2)	-	Chalcidoidea (19)	*Regiella insecticola* (88)^a^	Ascomycota (13)
	*Coleomegilla maculata* (57)			*Aphidiinae (20)	*Rickettsia* spp. (13)	Spermatophyta (1)
	*Harmonia axyridis* (27)				*Wolbachia* sp. (1)	
	*Spodoptera frugiperda* (18)				*Serratia* spp. (8,013)^b^	
	*Orius insidiosus* (15)					
	*Coccinella septempunctata* (11)					
	*Euschistus* sp. (6)					
	*Helicoverpa* sp. (6)					
*Harmonia axyridis*	*Doru luteipes* (8)	*Aphis* sp. (2)	*Aphis gossypii* (5)	Chalcidoidea (125)	*Regiella insecticola* (233)^a^	Ascomycota (3)
	*Aphis gossypii* (5)			*Aphidiinae (35)	*Hamiltonella* sp. (29)^a^	Spermatophyta (1)
					*Rickettsiella* sp. (12)	Streptophyta (1)
					*Spiroplasma* spp. (12)	
					*Wolbachia* sp. (12)	
					*Arsenophonus* sp. (6)	
					*Serratia* spp. (16,814)^b^	
					*Serratia symbiotica* (5)^a^	
*Doru luteipes*	*Plutella xylostella* (16)	*Plutella xylostella* (1)	-	*Aphidius sp. (1)	*Spiroplasma* spp. (1,419)	Spermatophyta (128)
	*Harmonia axyridis* (7)				*Rickettsia* spp. (642)	Streptophyta (2)
	Aphididae (2)				*Wolbachia* sp. (640)	Ascomycota (570)
					*Nosema* spp. (263)	Basidiomycota (7)
					*Serratia* spp. (11,095)^b^	
					*Serratia symbiotica* (29)^a^	
					*Regiella insecticola* (21)^a^	
					*Arsenophonus* sp. (9)	
					*Blattabacterium* spp. (6)	
					*Hamiltonella* sp. (3)^a^	
Control^c^	0	0	0	0	*Serratia* spp. (12,450)^b^	0

^a^ Aphid parasitoids and aphid symbionts.

^b^ *Serratia* sp. is presumably *S*. *marcescens*, an ubiquitous and highly abundant bacterium on earth, which was included in the bacteria genome database, although not strictly considered a symbiont. Curiously, this species was not detected in the predator *Cy*. *sanguinea*.

^c^ Recently emerged *H*. *axyridis* adults without feeding.

The most common identified prey were intraguild prey and aphids. Evidence of intraguild predation was detected in all predators. *Harmonia axyridis* was preyed upon by all other predators, whereas predation on *Cy*. *sanguinea* and *Hi*. *convergens* was not detected. *Hippodamia convergens* had the highest intraguild prey richness of three coccinellid species and one non-coccinellid predator (*Orius insidiosus*). Other extraguild prey detected were the lepidopterans *Spodoptera frugiperda*, *Helicoverpa* sp., and *Plutella xylostella*, the parasitoid *Di*. *coccinellae*, and the stink bug *Euschistus* sp. *Plutella xylostella* had a population outbreak in the organic cabbage field where *D*. *luteipes* was collected.

Non-prey foreign DNA was detected using the parasitoid, nuclear rRNA, and bacteria genome databases. Parasitoids were detected in all predators using the parasitoid reference database. However, at the 99% identity threshold used here, the reads matched the conserved V5 region of 18S rRNA that was identical in closely related species. Consequently, we reported the taxonomic level at which the match provided a reliable taxonomic determination (families of Chalcidoidea and Aphidiinae in Braconidae). For aphid parasitoids, this was sufficient to distinguish hyperparasitoids from primary parasitoids and to identify two broad groups of primary parasitoids. Chalcidoidea parasitoids were detected in all coccinellids, while Aphidiinae were detected in all but *Cy*. *sanguinea*.

The nuclear rRNA database revealed that all of the predators harbored a remarkable diversity of foreign DNA (Table 2). Reads matching Spermatophyta and fungal species (Streptophyta, Ascomycota, and Basidiomycota) were found in all of the species, but the dermapteran *D*. *luteipes* had by far the highest number of these reads (Table 2). The abundance of plant DNA may be from indirect consumption because *P*. *xylostella* caterpillars are typically stuffed with largely undigested leaf tissue in their guts. The large quantity of fungal DNA may be associated with the scavenging behavior of this species.

The bacteria genome database returned several bacterial species associated with insects, including facultative and obligate symbionts of aphids. Three aphid-specific symbionts, *Hamiltonella*, *R*. *insecticola*, and *S*. *symbiotica*, were detected in all four predators, but the strict obligate *Buchnera*, which is a powerful marker of aphid consumption immediately after feeding [31], was not detected in any of the samples. This probably happened because *Buchnera* DNA detection drops very fast after aphid consumption, while the other prey symbionts keep in the predator guts longer [31]. *Doru luteipes* was the only predator containing numerous reads of *Blattabacterium*, which is a mutualistic endosymbiont of cockroaches and some termites [40], and possibly other cryptic arthropods such as *Doru*. *Nosema*, an obligate parasitic microsporidia, was found only in *D*. *luteipes*.

### Predator qualitative trophic network

**Fig 1:**
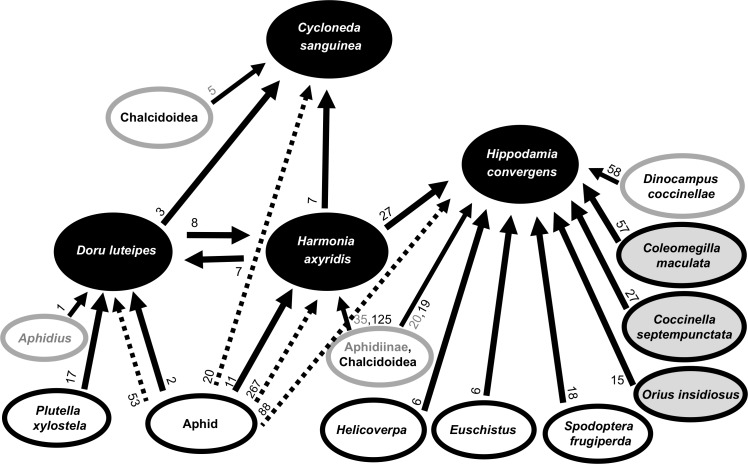
Qualitative trophic network for the focal predators using DNA shotgun-sequencing of their gut content. The focal predators are in black balloons, the prey are in white (extraguild) and grey (intraguild) balloons with black letters and edges, the parasitoids in white balloons with grey edges, in which grey letters are for the known aphid parasitoids, and black letters for other parasitoids. The arrows indicate the flux of biomass, in which black arrows indicate direct predation, and the dashed arrows indicate inferred predation associated with symbionts. The numbers at the origin of each arrow indicate the number of reads supporting the arrow, which for inferred predation is a thesum of the total aphid-specific symbiont reads.

Based on read matches, a qualitative trophic network was drawn for the four predator species in the Brazilian agroecosystem (Fig 1). Primary predation is straightforwardly established for the herbivorous and sap sucking species of Lepidoptera, Hemiptera and Homoptera. In some cases, the interaction could be established only indirectly through the presence of parasitoids or bacteria that establish an association with a primary predation event on aphids. For example, in *Cy*. *sanguinea*, even though no aphid DNA was detected, an aphid symbiont (*Hamiltonella*) was present. Similarly, an aphid parasitoid (Aphidiinae) and an aphid symbiont (*R*. *insecticola*) were found without detection of aphid DNA in *Hi*. *convergens*. In contrast, direct evidence for aphid DNA was seen in *H*. *axyridis* and *D*. *luteipes*, which also contained aphid parasitoid DNA (Aphidiinae and *Aphidius* sp.) and aphid symbionts (*R*. *insecticola*, *Hamiltonella* and *S*. *symbiotica*). Both species also harbored *Arsenophonus*, *Rickettsiella*, *Spiroplasma* and *Wolbachia*, of which some may be (endo)symbionts of the predators.

In sum, these foreign DNA detections can be integrated into a network of interactions of predation and parasitism, based on existing biological knowledge about the detected taxa for the indirectly established interactions (Fig 1). The interpretation of the detection of known parasitoids could signal both the direct ingestion by predators or the indirect ingestion of the parasitized hosts, or could even signal the parasitism of the predator itself in the case of the coccinellid specific parasitoid, *Di*. *coccinellae*. The total insect trophic links identified for each focal predator using the Insecta mitogenome, *cox1* and aphid genome databases was: two (plus one indirect link) for *Cy*. *sanguinea*, eight (plus one indirect) for *Hi*. *convergens*, two for *H*. *axyridis*, and three for *D*. *luteipes*, of which 93% were species or genus specific (Table 2).

## Discussion

The shotgun-sequencing of the DNA in the gut of predators enabled the identification of a set of taxonomically and functionally diverse foreign taxa, and this information was used to infer a qualitative trophic interaction network (Fig 1). We detected numerous foreign DNA sequences that could be identified to 15 taxa at the species or genus levels and assigned a clear trophic linkage. The results provided similar taxonomic breadth but higher taxonomic resolution for prey identification compared to other works using multiplex-PCR or metabarcoding on insect gut contents (Table 3). For most components of the trophic network these interactions were established based on just a few reads, although in some situations multiple evidences (e.g. co-detection of aphids and their microbiome) support the interaction. Particularly interesting are predator-on-predator interactions, inferred from the presence of predator-associated reads in a library generated from the gut of a different predator. However, some concerns remain about the minimum number of reads needed to confidently identify a prey species, because low number of reads can indicate not just low biomass of prey ingested or longer elapsed time after predation, but also false detection of spurious reads generated from sample contamination, sequencing or bioinformatics errors [41]. Although our negative control indicated lack of sample cross-contamination in our results, we nevertheless consider the network as drawn (Fig 1) to be no more than a heuristic summary of trophic interactions that require further testing with improved methodology and additional field sampling.

**Table 3:** Taxonomic Breadth and Resolution of the Foreign Species Identified in Arthropod Gut Contents by DNA-based Molecular Tools.

Arthropod taxa	*n*	Food/ prey	Breadth (food items/ táxon)	Resolution (% identified to species or genus)	Method	Target	Prey assignment			Reference
							Reference db	Read recovery	Filtering	
*Pterostichus melanarius*	50	10 taxa, slugs, worms, weevils, aphids	7.0	71	multiplex-PCR	*cox1*	--	--	--	[42]
Polyphagous grasshoppers (faeces samples)	3	Plants	3.0	44	metabarcoding, 454	P6 loop of chloroplast *tru*L intron	GenBank	MegaBLAST, no discussion of method for taxon assignment	20–85 bp, % identity manually curated, < 4 reads discarded	[43]
8 ground predator species	71.5 (range 6–155)	Arthropods in banana	3.6	86	metabarcoding, 454	mini *cox1* (127bp)	15 species sequenced and 20 species from GenBank after BLAST with raw 454 sequences	BLAST+, Nearest Neighbor algorithm	≥ 120 bp, 85% identity, < 2 reads discarded	[44]
4 foliar predators	5.75 (range 1–10)	Insects in agricultural fields	3.8	93	shotgun-sequencing, Illumina MiSeq	mito and nuclear DNA	Insecta mtDNA, *cox1* and aphid genomes	BLASTn and MegaBLAST, manually curated	≥ 225 bp, 98 to 99% identity	This paper

*n* = number of individual predators or faeces samples.

Biological knowledge about the identified taxa was required to establish the ingestion of aphids indirectly via aphid symbionts and parasitoids, and the ecological role of the linkages with parasitoids remains uncertain (direct or indirect consumption). Taken at face value, the identified linkages reveal predation on prey species known to occur at the sampled sites. *Cycloneda sanguinea* and *Hi*. *convergens* were the most abundant coccinellid species in the sampled organic farms. Although aphid DNA was not detected in either *Cy*. *sanguinea* or *Hi*. *convergens*, the detection of aphid parasitoid taxa in *Hi*. *convergens*, and aphid symbionts in both coccinellids strongly implies the consumption of aphids (Table 2). As the dominant aphid species in these habitats were probably *Uroleucon* sp. and *Brevicoryne* sp. [45], neither of which were in the aphid genome or mitogenome reference databases, the probability of detection of aphid DNA was reduced. In the dermapteran *D*. *luteipes* the observed direct consumption of aphids was also confirmed by the indirect linkages of an *Aphidius* parasitoid and three aphid-specific symbionts.

Intraguild predation was commonly detected and was asymmetrical. *Hippodamia convergens* fed on three other ladybirds, while no other predator species was detected in the gut of *H*. *axyridis*. In the temperate zone, *H*. *axyridis* is considered to be an aggressive intraguild predator and less commonly an intraguild prey [46]. However, in this Brazilian system, it was commonly detected as the intraguild prey. Finally, the detection of the coccinellid parasitoid, *Di*. *coccinellae*, represents an interesting addition to the trophic network with its potential to regulate the species composition of coccinellid assemblages. While we can plausibly assume that the Chalcidoidea and Aphidiinae parasitoids and symbionts of aphids (Table 2) were indirectly preyed upon through predation on their hosts, rather than direct predation on the free-living adults, *Di*. *coccinellae* pupates outside its host, and thus can be acquired by direct predation, rather than by feeding on a parasitized immature or adult coccinellid. The latter scenario seems unlikely because the parasitoid prefers adults [47], and intraguild predation is not common on adults [48]. A third scenario is that one of the ladybird specimens used for the gut analysis was itself parasitized by *Di*. *coccinellae*, and traces of its non-degraded DNA (from live tissue) was transferred into the gut extraction.

The key step of the DNA shotgun-sequencing analysis is the identification of reads as being foreign to the focal predator genome, which is established based on a stringent match to known barcode and genome sequences in the reference databases. For this, important issues are the completeness of identifying foreign DNA (reducing false negatives, i.e., identifying all of the foreign DNA in the predator gut) and the accurate taxonomic assignments of these DNA sequences (reducing false positives, i.e., not misidentifying taxa).

Regarding the issue of false negative risks, the foreign reads have to be detected among millions of others that are unidentified and presumably mostly originate from the predator nuclear genome. Thus, the taxonomic breadth and resolution of foreign DNA detection is directly related to the comprehensiveness of the DNA reference databases and the diagnostic power of the available genetic markers. Barcode and rRNA data were available in our reference databases for tens of thousands of species, but this may correspond to just a few percent of the total species in existence [49]. Thus, many species represented in the shotgun read mixture may remain unrecognized for the lack of representation in the reference database. Genetic variation and highly degraded prey DNA also add to the probability of missing a species represented in the mixture.

The use of the much more comprehensive *cox1* database with 58,367 Insecta species reference sequences did not detect more or unique prey species compared to the mitogenome database (Table 3). This database differs from the mitogenome database in its much greater species representation, but unlike the mitogenome database it was not supplemented with the local pool and this may result in recovery of fewer prey species. Secondly, the ~20x shorter length of the *cox1* barcode compared to mitogenome likely resulted in a lower recovery probability. A greater taxonomic coverage of mitogenomes is expected to aid in particular the resolution of parasitoid identifications and the detection of more aphid reads. Parasitoid detection was mainly against the parasitoid database, a collection of widely sequenced genes for diverse aphid parasitoid species, but the reads only matched the conserved regions of the 18S rRNA gene, which were invariable across entire clades and so parasitoid identifications could not be at the species level (Table 2).

The underrepresentation of mitochondrial and nuclear genomes of the aphid and parasitoid species in the DNA reference databases clearly limits the taxonomic resolution and the number of reads recovered. Where nuclear genome sequences available, the detection of a prey is expected with higher read numbers. For example, Paula *et al*. [31] detected *Acyrthosiphon pisum* in feeding trials using its nuclear genome at ~50x higher read numbers than by using the mitogenome. The predator gut content DNA datasets obtained here can be reanalyzed using a more comprehensive set of DNA reference databases to improve the taxonomic breadth and resolution of prey and other foreign species. In particular, investments in the construction of local mitogenome and draft nuclear genome databases of the species that share the habitat with the predators are needed because the single-locus *cox1* barcode database provides comparatively low read coverage for read matching (Table 2).

Regarding the risk of false positives, this is reduced because a high stringency of overlap length and sequence identity for taxonomic identification was applied, although this approach resulted in a low number of matching reads. For example, when we filtered the *Hi*. *convergens* gut content dataset against the Insecta mitogenome database using 90% identity, we obtained 775 reads for 11 foreign species, instead of 198 reads for eight foreign species when using 98% identity (Table 1). In the three species eliminated, all of the reads mapped to the mitochondrial D-loop region. Most of the remaining eliminated reads also mapped to this region. The proportion of D-loop matching reads was unexpectedly high at a 90% identity threshold, and does not give confidence in a prey species assignment. The D-loop matches are probably spurious, perhaps resulting from the high AT content and possibly conserved tandem repeats characteristic of these regions that limit the power for species discrimination. Therefore, the 98% identity threshold for prey identification in the mitogenome database was appropriate to eliminate false positive identifications. In a similar way, we establish identity thresholds for the other reference databases.

Possible ways to address the issue of low number of matching reads could be increasing the sequencing depth and optimizing the library insert size. In our various libraries we used insert sizes between 300 and 900 bp and paired-end sequencing in some cases, but while this produces high-quality reads for secure species-level identification, it probably reduces the number of reads to be detected for the prey DNA in digestion in the gut of the predator. Degraded DNA in the gut is expected to be short (e.g., ≤200 bp [50,51]) depending of the elapsed time since ingestion. Consequently, long library insert size may discriminate against the ingested DNA in favor of the non-degraded DNA from live predator tissue. At the same time, such an insert size can be used to generate longer sequences, which improves the accuracy of taxon identifications. Therefore, a balance between the library insert size and the read length should be considered. Much higher sequencing depth can be achieved at the same cost with single-end sequencing and shorter reads, and using the more powerful HiSeq platforms.

The metabarcoding approach may still be considered, due to the much larger number of foreign reads. However, PCR on degraded DNA is notoriously difficult, and variable PCR efficiency may skew the relative read numbers in the mixture due to imperfect primer target match [13,15,26, 52, 53]. Thus, metabarcoding does not necessarily improve the completeness of species detection [12, 54, 55] for trophic interaction studies of generalist predators. Clarke *et al*. [17] observed *in silico* that a set of widely used ‘generic arthropod’ *cox1* metabarcoding primers only managed to recover 43 to 64% of the species in a known mixture of arthropod DNA. Ji *et al*. [56] found that many *cox1* metabarcoding identifications are reliable, but were only able to link the most commonly recovered foreign sequences to operational taxonomic units (OTUs), despite the large *cox1* reference database. In addition, some of the reads can originate from contamination or chimeras [13, 57]. Metabarcoding studies therefore use an arbitrary threshold for the minimum number of reads per sample (e.g., discarding reads occurring less than four times, as in [42]).

In conclusion, even with modest sampling effort for four arthropod generalist predators, this DNA shotgun-sequencing approach provided similar taxonomic breadth and higher taxonomic resolution for prey identification compared to other works using multiplex-PCR or metabarcoding on insect gut contents (Table 3). At the current stage, however, the number of matching reads needs to be increased and the interpretation of low read matches needs to be validated. Despite this caveat, it is reasonable to conclude that shotgun-sequencing has significant advantages compared to the current methodologies and therefore might emerge as a preferred choice for gut content analysis because of: a) high confidence in the foreign DNA identifications; b) potential to simultaneously provide high taxonomic breadth and resolution; c) its practicality for working even with closely related prey and predator species, i.e. arthropods preyed upon by arthropods and even within a single guild; d) its barcode-free and PCR-free detection, eliminating PCR bias in species detection and measures of abundance; and e) an analysis of a broader spectrum of the ecosystem, including microbial symbionts and commensals, without the need for additional PCR targeting these groups.

## Supporting Information

S1 File Mitochondrial Insecta reference database, baits, elucidated mtDNA, bioinformatics pipeline, and mapping of reads to prey mitogenomes. (PDF)
