# Supplementary material for: Uncovering Trophic Interactions in Arthropod Predators through DNA Shotgun-Sequencing of Gut Contents
Source: PLoS One. 2016 Sep 13;11(9):e0161841. doi: 10.1371/journal.pone.0161841 (PMC5021305; doi:10.1371/journal.pone.0161841)
Supplement: S1 File — (PDF) [file pone.0161841.s001.pdf]

S1 File (Supporting Information)

Uncovering trophic interactions in arthropod predators using DNA shotgun-sequencing of gut contents

Débora P. Paula, Benjamin Linard, Alex Crampton-Platt, Amrita Srivathsan, Martijn J. T. N. Timmermans, Edison R. Sujii, Carmen S. S. Pires, Lucas M. Souza, David A. Andow and Alfried P. Vogler

Mitochondrial Insecta reference database, baits, elucidated mtDNA, bioinformatics pipeline, and mapping of reads to prey mitogenomes

Table A. Partial and complete insect species mitochondrial DNA used in the mitogenome reference database.

Order	Family	Species	GenBank ID	Size (bp)
Archaeognatha	Machilidae	*Pedetontus silvestrii*	gi|218157371|ref|NC_011717.1|	15879
		Petrobius brevistylis	gi|84488608|ref|NC_007688.1|	15698
		Songmachilis xinxiangensis	gi|511347795|ref|NC_021384|	15473
		Trigoniophthalmus alternatus	gi|171473585|ref|NC_010532.1|	16197
	Meinertellidae	*Nesomachilis australica*	gi|62161388|ref|NC_006895.1|	15474
Blattaria	Blattellidae	*Blattella bisignata*	gi|403531634|ref|NC_018549.1|	16470
	Cryptocercidae	*Cryptocercus relictus*	gi|394831453|ref|NC_018132.1|	15373
	Mastotermitidae	*Mastotermes darwiniensis*	gi|394831207|ref|NC_018120.1|	15487
	Rhinotermitidae	*Reticulitermes flavipes*	gi|148368751|ref|NC_009498.1|	16565
		Reticulitermes santonensis	gi|148368807|ref|NC_009499.1|	16567
Blattodea	Blattellidae	*Blattella germanica*	gi|242624230|ref|NC_012901.1|	15025
		Periplaneta americana	gi|378829200|ref|NC_016956.1|	15584
		Periplaneta fuliginosa	gi|50812103|ref|NC_006076.1|	14996
	Corydiidae	*Eupolyphaga sinensis*	gi|299827802|ref|NC_014274.1|	15553
Coleoptera	Anobiidae	*Xyletinus* sp.	gi|402535264|gb|JX313671	9719
	Anthicidae	*Ischalia* sp.	gi|309253244|gb HQ232827.1|	12396
		Omonadus floralis	gi|309253219|gbHQ232825.1|	12235
		Platystomos albinus	gi|347950302|gb|JN163968|	10594
	Attelabidae	*Apoderus coryli*	gi|347950276|gb|JN163966|	9899
		Deporaus betulae	gi|347950017|gb|JN163945|	10673
		Byctiscus populi	gi|441349239|ref|NC_018760|	9468
	Aspidytidae	*Aspidytes niobe*	gi|224587990|ref|NC_012139.1|	14257

Attelabidae	*Byctiscus populi*	gi|441349239|ref|NC_018760.1|	9468
Boridae	Boros schneideri	gi309253197HQ232823.1	12319
Bostrichidae	*Apatides fortis*	gi|270267691|ref|NC_013582.1|	16171
Bothriderida	*Teredus cylindricus*	gi|402535420|gb|JX313684	5005
Brachyceridae	*Brachycerus muricatus*	gi|347950328|gb|JN163970	10817
Brentidae	*Nanophyes marmoratus*	gi|347950030|gb|JN163946	10629
	Rhopalapion longirostre	gi|347950289|gb|JN163967	11152
Buprestidae	*Acmaeodera* sp.	gi|270267663|ref|NC_013580.1|	16217
Byrrhoidae	*Byrrhus* sp.	gi|374639339|gb|JQ034419	10625
	Chrysochroa fulgidissima	gi|238866876|ref|NC_012765.1|	15592
Cantharidae	*Cantharis pellucida*	gi309253146HQ232817.1	12400
	Chauliognathus opacus	gi|270267607|ref|NC_013576.1|	14893
Carabidae	*Calosoma* sp.	gi|400201477|ref|NC_018339.1|	16462
	Damaster mirabilissimus	gi|364284050|ref|NC_016469.1|	16823
	Promecognathus crassus	gi|402535192|gb|JX313665	16535
	Trechini sp.	gi398660193gbHQ232802.2	11460
Cerambycidae	*Anoplophora glabripennis*	gi|109689623|ref|NC_008221.1|	15774
	Batocera lineolata	gi556505458refNC_022671.1	15418
	Closteromerus claviger	gi309253017gbHQ232804.1	12185
	Disteniazteca fimbriata	gi|407911488|gb|JX221000	9776
	Monochamus alternatus	gi|428673748|gb|JX987292	14649
	Necydalis ulmi	gi|407911348|gb|JX220989	10656
	Prioninae sp.	gi|407911374|gb|JX220991	10679
	Psacothea hilaris	gi|255506410|ref|NC_013070.1|	15856
	Stenurella nigra	gi|407911463|gb|JX220998	10617
	Vesperus conicicollis	gi|407911437|gb|JX220996	10682
Cerylonidae	*Cerylon histeroides*	gi398660291gbHQ232821.2	9694
Chaetosomatidae	*Chaetosoma scaritides*	gi|208433946|ref|NC_011324.1|	15511
Chrysomelidae	*Arescus labiatus*	gi|407911335|gb|JX220988	10511
	Crioceris duodecimpunctata	gi|18390112|ref|NC_003372.1|	15880
	Donacia distincta	gi|407911476|gb|JX220999	9790
	Exema canadensis	gi|407911361|gb|JX220990	11305
	Imatidium capense	gi|407911400|gb|JX220993	9653
	Orsodacne lineola	gi|407911412|gb|JX220994	9828
	Peploptera acromialis	gi309253065gbHQ232809.1	12549
	Phaedon armoraciae	gi|407911387|gb|JX220992	10645
	Spilopyra sumptuosa	gi|407911450|gb|JX220997	10170
Coccinellidae	*Coccinella septempunctata*	gi|375066069|gb|JQ321839.1|	18965
Cucujidae	*Cucujus clavipes*	gi|400201619|ref|NC_018350.1|	15642
Curculionidae	*Anthonomus pomorum*	gi|347950095|gb|JN163951	10606
	Barynotus obscurus	gi|408772082|ref|NC_018756|	10016
	Brachytemnus porcatus	gi|408772095|ref|NC_018758|	10666
	Ceutorhynchus assimilis	gi|347950158|gb|JN163956	11346
	Cionus olens	gi|347950184|gb|JN163958	10702

	Hylobitelus xiaoi	gi556505585refNC_022680.1	16123
	Hylobius abietis	gi|347950133|gb|JN163954	10673
	Hypera postica	gi|347950120|gb|JN163953	10646
	Ips cembrae	gi|347950220|gb|JN163961	5670
	Kyklioacalles aubei	gi|347950171|gb|JN163957	11168
	Larinus turbinatus	gi|347950108|gb|JN163952	9809
	Liophloeus tessulatus	gi|347950043|gb|JN163947	10628
	Melanobaris laticollis	gi|441349169|ref|NC_018757|	10792
	Otiorhynchus rugosostriatus	gi|347950315|gb|JN163969	10675
	Platypus cylindrus	gi|347950239|gb|JN163963	10583
	Polydrusus marginatus	gi|336285677|gb|JN039360	10692
	Scolytus sp.	gi|441349209|ref|NC_018759.1|	10567
	Sitona lineatus	gi|347950056|gb|JN163948	10720
	Sitophilus granarius	gi|347950197|gb|JN163959	6291
	Sphenophorus sp.	gi|400201633|ref|NC_018351.1|	15052
	Strophosoma melanogrammum	gi|347950069|gb|JN163949	10511
Dascillidae	*Dascillus cervinus*	gi|377823014|ref|NC_016919.1|	10662
Dermestidae	*Anthrenus* sp.	gi398660231gbHQ232808.2	12847
	Dermestes peruvianus	gi|402535408|gb|JX313683	9756
	Trinodes hirtus	gi|402535180|gb|JX313664	9780
Drilidae	*Drilus flavescens*	gi309253125gbHQ232815.1	12519
Dytiscidae	*Copelatus* sp.	gi|407911424|gb|JX220995	10645
	Hydroporus palustris	gi|402535255|gb|JX313670	8114
Elateridae	*Pyrophorus divergens*	gi|159159429|ref|NC_009964.1|	16120
Elmidae	*Elmidae* sp.	gi309253075gb HQ232810.1	12409
Endomychidae	*Endomychus coccineus*	gi|402535218|gb|JX313667	9695
Erotylidae	*Tritoma bipustulata*	gi398660296gbHQ232822.2	8801
Eucnemidae	*Melasis buprestoides*	gi398660258gbHQ232813.2	6625
Eulichadidae	*Eulichas* sp.	gi309253092bgHQ232812.1	10683
Glaphyridae	*Glaphyrus comosus*	gi|402535288|gb|JX313673	10636
Gyrinidae	*Macrogyrus oblongus*	gi|258649546|ref|NC_013249.1|	16643
Haliplidae	*Haliplus flavicollis*	gi|402535338|gb|JX313677	13612
	Peltodytes quadratus	gi|402535427|gb|JX313685	9819
Heteroceridae	*Heterocerus fenestratus*	gi309253085gbHQ232811.1	12329
Histeridae	*Abraeinae* sp.	gi|402535484|gb|JX313690	9758
	Euspilotus scissus	gi|400201661|ref|NC_018353.1|	14763
Hydraenidae	*Hydraena* sp.	gi 309252979gb HQ232800.1	5792
Hydrochidae	*Hydrochus* sp.	gi 309252987gb HQ232801.1	12330
Hydrophilidae	*Tropisternus* sp.	gi|400201605|ref|NC_018349.1|	16372
Hydroscaphidae	*Hydroscapha granulum*	gi|224588060|ref|NC_012144.1|	15975
Kateretidae	*Kateretes pedicularius*	gi|402535472|gb|JX313689	9786
Lampyridae	*Drilaster* sp.	gi309253135gbHQ232816.1	9683
	Pyrocoelia rufa	gi|21326209|ref|NC_003970.1|	17739
Latrididae	*Enicmus brevicornis*	gi|402535383|gb|JX313681	9787

	Leiodidae	*Nargus velox*	gi|402535301|gb|JX313674	9771
	Limnichidae	*Limnichidae* sp.	gi|374639319|gb|JQ034416	14388
	Lucanidae	*Lucanidae* sp.	gi|402535230|gb|JX313668	10605
		Lucanus mazama	gi|270267635|ref|NC_013578.1|	15261
	Lycidae	*Lycus dentipes*	gi309253113gbHQ232814.1	12257
	Lymexylidae	*Hylecoetus dermestoides*	gi309253168gbHQ232820.1	11236
	Melandryidae	*Osphya bipunctata*	gi|402535313|gb|JX313675	9919
	Meloidae	*Meloidae* sp.	gi|302632997|gb|HM486073	10589
	Meryidae	*Meru phyllisae*	gi|402535496|gb|JX313691	10639
	Mordellidae	*Mordella atrata*	gi|258649600|ref|NC_013254.1|	15540
	Mycetophagidae	*Mycetophagus quadripustulatus*	gi309253207gbHQ232824.1	12281
	Nemonychidae	*Doydirhynchus austriacus*	gi|347950252|gb|JN163964	10666
	Noteridae	*Noteridae* sp.	gi|402535206|gb|JX313666	10038
	Oedemeridae	*Oedemera virescens*	gi309253231gbHQ232826.1	10653
	Ommatidae	*Tetraphalerus bruchi*	gi|208433960|ref|NC_011328.1|	15689
	Phalacridae	*Phalacridae* sp.	gi398660198gbHQ232803.2	12783
	Priasilphidae	*Priasilpha obscura*	gi|208433773|ref|NC_011326.1|	16603
	Ptinidae	*Ptinus rufipes*	gi398660281gbHQ232819.2	10458
	Rhagophthalmidae	*Rhagophthalmus lufengensis*	gi|190349565|ref|NC_010969.1|	15982
		Rhagophthalmus ohbai	gi|192293811|ref|NC_010964.1|	15704
	Salpingidae	*Salpingus aeneus*	gi|402535376|gb|JX313680	6045
	Scarabaeidae	*Rhopaea magnicornis*	gi|258650138|ref|NC_013252.1|	17522
	Scraptiidae	*Anaspis* sp.	gi398660213gbHQ232806.2	10622
	Scirtidae	*Cyphon* sp.	gi|208433918|ref|NC_011320.1|	15919
	Silphidae	*Necrophila americana*	gi|400201647|ref|NC_018352.1|	16902
	Silvanidae	*Silvanus unidentatus*	gi|402535452|gb|JX313687	6927
	Sphaeriusidae	*Sphaerius* sp.	gi|208433932|ref|NC_011322.1|	15121
	Staphylinidae	*Hypomedon debilicornis*	gi|402535276|gb|JX313672	9850
		Parocyusa longitarsis	gi|402535352|gb|JX313678	9785
	Tenebrionidae	*Adelium* sp.	gi|270267294|ref|NC_013554.1|	16449
		Eutrapela ruficollis	gi398660207gbHQ232805.2	12545
		Tribolium castaneum	gi|133755325|ref|NC_003081.2|	15881
	Trachypachidae	*Trachypachus holmbergi*	gi|208433759|ref|NC_011329.1|	15722
	Zeugophoridae	*Zeugophora* sp.	gi309253046gbHQ232807.1	10656
Dermaptera	Pygidicranidae	*Challia fletcheri*	gi|403531341|ref|NC_018538.1|	20456
Diptera	Agromyzidae	*Liriomyza bryoniae*	gi|372291921|ref|NC_016713.1|	16183
		Liriomyza huidobrensis	gi|372291963|ref|NC_016716.1|	16236
		Liriomyza sativae	gi|343198283|ref|NC_015926.1|	15551
		Liriomyza trifolii	gi|299829005|ref|NC_014283.1|	16141
	Anisopodidae	*Sylvicola fenestralis*	gi|357017867|ref|NC_016176.1|	16234
	Caliphoridae	*Calliphora vicina*	gi|426406492|ref|NC_019639.1|	16112
		Chrysomya albiceps	gi|426406380|ref|NC_019631.1|	15491
		Chrysomya bezziana	gi|426406394|ref|NC_019632.1|	15236

	Chrysomya megacephala	gi|426406408|ref|NC_019633.1|	15273
	Chrysomya putoria	gi|13435200|ref|NC_002697.1|	15837
	Chrysomya rufifacies	gi|426406422|ref|NC_019634.1|	15412
	Chrysomya saffranea	gi|426406436|ref|NC_019635.1|	15839
	Cochliomyia hominivorax	gi|12711796|ref|NC_002660.1|	16022
	Hemipyrellia ligurriens	gi|426406478|ref|NC_019638.1|	15938
	Lucilia cuprina	gi|426580980|ref|NC_019573.1|	15952
	Lucilia porphyrina	gi|426406464|ref|NC_019637.1|	15877
	Lucilia sericata	gi|154800398|ref|NC_009733.1|	15945
	Pollenia rudi	gi|409031945|gb|JX913761	14340
	Protophormia terraenovae	gi|426406450|ref|NC_019636.1|	15170
Cecidomyiidae	*Asphondylia rosetta*	gi|254763506|gb|GQ387650	11938
	Mayetiola destructor	gi|255506277|ref|NC_013066.1|	14759
	Rhopalomyia pomum	gi|255506236|ref|NC_013063.1|	14503
Ceratopogonidae	*Culicoides arakawae*	gi|157326160|ref|NC_009809.1|	18135
Chironomidae	*Chironomus tepperi*	gi|357017769|ref|NC_016167.1|	15652
Culicidae	*Aedes aegypti*	gi|164523399|ref|NC_010241.1|	16655
	Aedes albopictus	gi|58372083|ref|NC_006817.1|	16665
	Anopheles albitarsis	gi|470231415|ref|NC_020662.1|	15413
	Anopheles cracens	gi|470235772|ref|NC_020768.1|	15412
	Anopheles darlingi	gi|299828908|ref|NC_014275.1|	15386
	Anopheles deaneorum	gi|470231429|ref|NC_020663.1|	15424
	Anopheles dirus	gi|398314347|gb|JX219732	15404
	Anopheles farauti	gi|471193906|ref|NC_020770.1|	15412
	Anopheles funestus	gi|107736148|ref|NC_008070.1|	15354
	Anopheles gambiae	gi|5834911|ref|NC_002084.1|	15363
	Anopheles hinesorum	gi|470235786|ref|NC_020769.1|	15336
	Anopheles koliensis	gi|398314487|gb|JX219742	15412
	Anopheles punctulatus	gi|398314417|gb|JX219737	15412
	Anopheles quadrimaculatus	gi|5835918|ref|NC_000875.1|	15455
	Culex pipiens pipiens	gi|322422420|ref|NC_015079.1|	14856
	Culex quinquefasciatus	gi|308745776|ref|NC_014574.1|	15587
Drosophilidae	*Drosophila littoralis*	gi|215259920|ref|NC_011596.1|	16017
	Drosophila mauritiana	gi|45332696|ref|NC_005779.1|	14964
	Drosophila melanogaster	gi|5835233|ref|NC_001709.1|	19517
	Drosophila pseudoobscura	gi|400201591|ref|NC_018348.1|	14914
	Drosophila sechellia	gi|45332682|ref|NC_005780.1|	14950
	Drosophila simulans	gi|45332829|ref|NC_005781.1|	14972
	Drosophila yakuba	gi|5834829|ref|NC_001322.1|	16019
Fergusoninidae	*Fergusonina taylori*	gi|375262305|ref|NC_016865.1|	16000
Muscidae	*Haematobia irritans irritans*	gi|67009990|ref|NC_007102.1|	16078
	Musca domestica	gi|158344510|gb|EU154477	14347
	Stomoxys calcitrans	gi|95105398|gb|DQ533708	16790
Mycetophilidae	*Arachnocampa flava*	gi|357018198|ref|NC_016204.1|	16923

	Nemestrinidae	*Trichophthalma punctata*	gi|120944055|ref|NC_008755.1|	16396
	Oestridae	*Dermatobia hominis*	gi|54306052|ref|NC_006378.1|	16360
		Hypoderma lineatum	gi|290967645|ref|NC_013932.1|	16354
	Pachyneuridae	*Cramptonomyia spenceri*	gi|357018184|ref|NC_016203.1|	16274
	Ptychopteridae	*Bittacomorphella fenderiana*	gi|356488917|gb|JN861745	15609
		Ptychoptera sp.	gi|357018156|ref|NC_016201.1|	15214
	Rutilia	*Rutilia goerlingiana*	gi|426406506|ref|NC_019640.1|	15331
	Sarcophagidae	*Sarcophaga impatiens*	gi|383931284|ref|NC_017605.1|	15169
	Sciaridae	*Bradysia amoena*	gi|254763519|gb|GQ387651	14049
	Syrphidae	*Simosyrphus grandicornis*	gi|120944038|ref|NC_008754.1|	16141
	Tabanidae	*Cydistomyia duplonotata*	gi|120944077|ref|NC_008756.1|	16247
		Elodia flavipalpis	gi|394831173|ref|NC_018118.1|	14932
		Exorista sorbillans	gi|312233479|ref|NC_014704.1|	14960
	Tanyderidae	*Protoplasa fitchii*	gi|357018170|ref|NC_016202.1|	16154
	Tephritidae	*Bactrocera carambolae*	gi|156765981|ref|NC_009772.1|	15915
		Bactrocera correcta	gi|408772432|ref|NC_018787.1|	15936
		Bactrocera cucurbitae	gi|351000070|ref|NC_016056.1|	15825
		Bactrocera dorsalis	gi|120586710|ref|NC_008748.1|	15389
		Bactrocera minax	gi|302632666|ref|NC_014402.1|	16043
		Bactrocera oleae	gi|41057423|ref|NC_005333.1|	15815
		Bactrocera papayae	gi|156765967|ref|NC_009770.1|	15915
		Bactrocera philippinensis	gi|156765995|ref|NC_009771.1|	15915
		Bactrocera tryoni	gi|309259980|ref|NC_014611.1|	15925
		Ceratitis capitata	gi|5835876|ref|NC_000857.1|	15980
		Procecidochares utilis	gi|459627023|ref|NC_020463.1|	15922
	Tipulidae	*Tipula abdominalis*	gi|356488889|gb|JN861743	14566
	Trichoceridae	*Paracladura trichoptera*	gi|357017825|ref|NC_016173.1|	16143
		Trichocera bimacula	gi|357017797|ref|NC_016169.1|	16140
Ephemeroptera	Ephemeridae	*Ephemera orientalis*	gi|229324789|ref|NC_012645.1|	16463
	Heptageniidae	*Parafronurus youi*	gi|209427686|ref|NC_011359.1|	15481
	Siphlonuridae	*Siphlonurus immanis*	gi|288903297|ref|NC_013822.1|	15529
Grylloblattodea	Grylloblattidae	*Grylloblatta sculleni*	gi|82792109|gb|DQ241796	15595
Hemiptera	Aleyrodidae	*Aleurochiton aceris*	gi|51830142|ref|NC_006160.1|	15388
		Aleurodicus dugesii	gi|49146478|ref|NC_005939.1|	15723
		Bemisia tabaci	gi|52220940|ref|NC_006279.1|	15322
		Neomaskellia andropogonis	gi|51830183|ref|NC_006159.1|	14496
		Tetraleurodes acaciae	gi|52221066|ref|NC_006292.1|	15080
		Trialeurodes vaporariorum	gi|52220968|ref|NC_006280.1|	18414
	Alydidae	*Riptortus pedestris*	gi|225697513|ref|NC_012462.1|	17191
	Anthocoridae	*Orius niger*	gi|225676739|ref|NC_012429.1|	14494
	Aphelocheiridae	*Aphelocheirus ellipsoideus*	gi|215788935|gb|FJ456939	14574
	Aphididae	*Acyrthosiphon pisum*	gi|213948225|ref|NC_011594.1|	16971
		Aphis glycines	gi|511369730|gb|KC840675	13002
		Cavariella salicicola	gi556505630refNC_022682.1	16317

	Diuraphis noxia	gi556506243refNC_022727.1	15784
	Pterocomma pilosum	gi|511369752|gb|KC840676	12529
	Schizaphis graminum	gi|51830049|ref|NC_006158.1|	15721
Aradidae	*Brachyrhynchus hsiaoi*	gi556505444refNC_022670.1	15250
	Neuroctenus parus	gi|225697511|ref|NC_012459.1|	15354
Belostomatidae	*Diplonychus rusticus*	gi|215788949|gb|FJ456940	14596
Berytidae	*Yemmalysus parallelus*	gi|225697515|ref|NC_012464.1|	15747
Cercopidae	*Abidama producta*	gi|339906376|ref|NC_015799.1|	15277
	Callitettix versicolor	gi|435856746|ref|NC_020031.1|	15374
	Paphnutius ruficeps	gi|482651017|ref|NC_021100.1|	14841
	Philaenus spumarius	gi|49146630|ref|NC_005944.1|	16324
Cicadellidae	*Homalodisca vitripennis*	gi|62161309|ref|NC_006899.1|	15304
Colobathristidae	*Phaenacantha marcida*	gi|225697512|ref|NC_012460.1|	14540
Coreidae	*Hydaropsis longirostris*	gi|225697508|ref|NC_012456.1|	16521
Corixidae	*Sigara septemlineata*	gi|215788963|gb|FJ456941	15724
Delphacidae	*Laodelphax striatellus*	gi|283099275|ref|NC_013706.1|	16513
	Nilaparvata lugens	gi|525340918|ref|NC_021748|	17619
	Sogatella furcifera	gi|511348229|ref|NC_021417|	16612
Dinidoridae	*Coridius chinensis*	gi|384597640|gb|JQ739179	14648
Enicocephalidae	*Stenopirates* sp.	gi|347600351|ref|NC_016017.1|	15384
Flatidae	*Geisha distinctissima*	gi|228015376|ref|NC_012617.1|	15971
Fulgoridae	*Laternaria candelaria*	gi|426405749|ref|NC_019576.1|	16021
	Lycorma delicatula	gi|240266556|ref|NC_012835.1|	15971
Gelastocoridae	*Nerthra* sp.	gi|240266730|ref|NC_012838.1|	16079
Gerridae	*Aquarius paludum*	gi|240266626|ref|NC_012841.1|	15380
Helotrephidae	*Helotrephes* sp.	gi|240266410|ref|NC_012822.1|	15130
Hydrometridae	*Hydrometra* sp.	gi|240266640|ref|NC_012842.1|	15416
Issidae	*Sivaloka damnosus*	gi|299829047|ref|NC_014286.1|	15287
Largidae	*Physopelta gutta*	gi|225676740|ref|NC_012432.1|	14935
Leptopodidae	*Leptopus* sp.	gi|215789033|gb|FJ456946	14516
Lygaeidae	*Geocoris pallidipennis*	gi|225676457|ref|NC_012424.1|	14592
Malcidae	*Chauliops fallax*	gi|470235814|ref|NC_020772.1|	15739
	Malcus inconspicuus	gi|225697510|ref|NC_012458.1|	15575
Margarodidae	*Sasakia charonda*	gi|297572359|ref|NC_014224.1|	15244
Miridae	*Nesidiocoris tenuis*	gi556505543refNC_022677.1	17544
Nabidae	*Alloeorhynchus bakeri*	gi|359422225|ref|NC_016432.1|	15851
	Gorpis annulatus	gi|426406015|ref|NC_019595.1|	16660
	Gorpis humeralis	gi|426405987|ref|NC_019593.1|	18165
	Himacerus apterus	gi|334884227|gb|JF927831	15995
	Himacerus nodipes	gi|334884241|gb|JF927832	16457
	Nabis apicalis	gi|426406001|ref|NC_019594.1|	15588
Nepidae	*Laccotrephes robustus*	gi|240266354|ref|NC_012817.1|	15321
Nepomorpha	*Ilyocoris cimicoides*	gi|240266682|ref|NC_012845.1|	14609
Notonectidae	*Enithares tibialis*	gi|240266368|ref|NC_012819.1|	15262

	Ochteridae	*Ochterus marginatus*	gi|240266382|ref|NC_012820.1|	14609
	Peloridiidae	*Hackeriella veitchi*	gi|452848498|ref|NC_020309.1|	15793
		Xenophyes cascu	gi|325975774|gb|JF323862	14117
	Pentatomidae	*Dolycoris baccarum*	gi|452849994|ref|NC_020373.1|	16549
		Eusthenes cupreus	gi546143635refNC_022449.1	16229
		Halyomorpha halys	gi|260150943|ref|NC_013272.1|	16518
		Lygus lineolaris	gi|529217642|ref|NC_021975|	17027
		Macroscytus subaeneus	gi|225697509|ref|NC_012457|	14620
		Nezara viridula	gi|218456815|ref|NC_011755.1|	16889
		Urochela quadrinotata	gi|442769758|ref|NC_020144.1|	16587
	Phylloxeridae	*Daktulosphaira vitifoliae*	gi|70779445|gb|DQ021446	12349
	Plataspidae	*Coptosoma bifaria*	gi|225697506|ref|NC_012449.1|	16179
	Plataspididae	*Megacopta cribraria*	gi|327492625|ref|NC_015342.1|	15647
	Pleidae	*Paraplea frontalis*	gi240266410refNC_012822.1	15130
	Psyllidae	*Pachypsylla venusta*	gi|51830063|ref|NC_006157.1|	14711
	Pyrrhocoridae	*Dysdercus cingulatus*	gi|225660332|ref|NC_012421.1|	16249
	Reduviidae	*Agriosphodrus dohrni*	gi|340001900|ref|NC_015842.1|	16470
		Sirthenea flavipes	gi|442769744|ref|NC_020143.1|	15961
		Triatoma dimidiata	gi|11182462|ref|NC_002609.1|	17019
		Valentia hoffmanni	gi|240266424|ref|NC_012823.1|	15625
	Rhopalidae	*Stictopleurus subviridis*	gi|242543728|ref|NC_012888.1|	15319
		Aeschyntelus notatus	gi|225697457|ref|NC_012446.1|	14532
	Ricaniidae	*Ricania marginalis*	gi|426406043|ref|NC_019597.1|	15698
	Saldidae	*Saldula arsenjevi*	gi|225697514|ref|NC_012463.1|	15324
Hymenoptera	Agonidae	*Ceratosolen solmsi*	gi|334191939|gb|JF816396	10044
		Philotrypesis sp.	gi|333946260|gb|JF808722	11997
		Philotrypesis pilosa	gi|333946274|gb|JF808723	15122
	Apidae	*Apis cerana*	gi|299829158|ref|NC_014295.1|	15895
		Apis florea	gi|511348005|ref|NC_021401|	17694
		Apis mellifera ligustica	gi|5834925|ref|NC_001566.1|	16343
		Bombus hypocrita sapporensis	gi|221143400|ref|NC_011923.1|	15468
		Bombus ignitus	gi|190349384|ref|NC_010967.1|	16434
		Melipona bicolor	gi|27733916|ref|NC_004529.1|	14422
	Bethylidae	*Cephalonomia gallicola*	gi|225690874|gb|FJ823227	16720
	Braconidae	*Aphidius gifuensis*	gi|281188260|gb|GU097658	11996
		Cotesia vestalis	gi|299827774|ref|NC_014272.1|	15543
		Diachasmimorpha longicaudata	gi|262073226|gb|GU097655	13850
		Macrocentrus camphoraphilus	gi|262073239|gb|GU097656	15801
		Meteorus pulchricornis	gi|281188258|gb|GU097657	10186
		Phanerotoma flava	gi|262073213|gb|GU097654	10171
		Spathius agrili	gi|299828950|ref|NC_014278.1|	15425
	Cephidae	*Cephus cinctus*	gi|237515443|ref|NC_012688.1|	19339
	Crabronidae	*Philanthus triangulum*	gi|380877155|ref|NC_017007.1|	16029
	Eumenidae	*Abispa ephippium*	gi|211908570|ref|NC_011520.1|	16953

	Evaniidae	*Evania appendigaster*	gi|258649392|ref|NC_013238.1|	17817
	Formicidae	*Camponotus chromaiodes*	gi|429535900|gb|JX966368	12473
		Pristomyrmex punctatus	gi|322422364|ref|NC_015075.1|	16180
		Solenopsis geminata	gi|312233080|ref|NC_014669.1|	15552
		Solenopsis invicta	gi|312233122|ref|NC_014672.1	15549
		Solenopsis richteri	gi|312233150|ref|NC_014677.1|	15560
	Ichneumonidae	*Diadegma semiclausum*	gi|237869070|ref|NC_012708.1|	18728
		Enicospilus sp.	gi|215789178|gb|FJ478177	15300
		Venturia canescens	gi|215789164|gb|FJ478176	13637
	Mutillidae	*Radoszkowskius oculata*	gi|306960063|ref|NC_014485.1|	18442
	Orussidae	*Orussus occidentalis*	gi|237515457|ref|NC_012689.1|	15947
	Pergidae	*Perga condei*	gi|55274303|gb|AY787816	13416
	Scelionidae	*Trissolcus basalis*	gi|357197259|gb|JN903532	15768
	Stephanidae	*Schlettererius cinctipes*	gi|215789151|gb|FJ478175	10984
	Tenthredinidae	*Monocellicampa pruni*	gi|409107525|gb|JX566509	15169
	Vanhorniidae	*Vanhornia eucnemidarum*	gi|114052840|ref|NC_008323.1|	16574
	Vespidae	*Polistes* sp.	gi|152031222|gb|EU024653	14741
Isoptera	Hodotermitidae	*Microhodotermes viator*	gi|394831241|ref|NC_018122.1|	15704
		Porotermes adamsoni	gi|394831226|ref|NC_018121.1|	16039
	Kalotermitidae	*Neotermes insularis*	gi|394831275|ref|NC_018124.1|	15799
	Rhinotermitidae	*Coptotermes formosanus*	gi|339906390|ref|NC_015800.1|	16326
		Coptotermes lacteus	gi|394831292|ref|NC_018125.1|	16326
		Heterotermes sp.	gi|394831375|ref|NC_018127.1|	16370
		Reticulitermes hageni	gi|148368779|ref|NC_009501.1|	16590
		Reticulitermes virginicus	gi|148368765|ref|NC_009500.1|	16513
		Schedorhinotermes breinli	gi|394831308|ref|NC_018126.1|	15864
	Termitidae	*Drepanotermes* sp.	gi|394831406|ref|NC_018129.1|	16542
		Macrognathotermes errator	gi|394831422|ref|NC_018130.1|	16330
		Macrotermes barneyi	gi|404333681|ref|NC_018599.1|	15940
		Macrotermes subhyalinus	gi|394831390|ref|NC_018128.1|	16351
		Nasutitermes triodiae	gi|394831436|ref|NC_018131.1|	15849
		Naupactus xanthographus	gi|400201675|ref|NC_018354.1|	15026
	Termopsidae	*Zootermopsis angusticollis*	gi|394831259|ref|NC_018123.1|	15483
Lepidoptera	Arctiidae	*Amata formosae*	gi|511348215|ref|NC_021416|	15463
		Hyphantria cunea	gi|295065650|ref|NC_014058.1|	15481
	Bombycidae	*Bombyx mandarina*	gi|18644896|ref|NC_003395.1|	15928
		Bombyx mori	gi|8572562|ref|NC_002355.1|	15643
		Rondotia menciana	gi|529217460|ref|NC_021962|	15301
	Crambidae	*Cnaphalocrocis medinalis*	gi|345894187|ref|NC_015985.1|	15388
		Diatraea saccharalis	gi|260150957|ref|NC_013274.1|	15490
		Dichocrocis punctiferalis	gi|511347851|ref|NC_021389|	15355
		Elophila interruptalis	gi|525341016|ref|NC_021756|	15351
		Glyphodes quadrimaculalis	gi556505873refNC_022699.1	15255
		Maruca vitrata	gi|308084372|gb|HM751150	14054

	Ostrinia nubilalis	gi|18314290|ref|NC_003367.1|	14535
	Paracymoriza prodigalis	gi|441403371|ref|NC_020094.1|	15326
	Scirpophaga incertulas	gi|511348173|ref|NC_021413|	15223
Ennominae	*Biston panterinaria*	gi|435855846|ref|NC_020004.1|	15517
Erebidae	*Lymantria dispar*	gi|242610072|ref|NC_012893.1|	15569
Geometridae	*Phthonandria atrilineata*	gi|170787328|ref|NC_010522.1|	15499
Glossata	*Chilo suppressalis*	gi|334362128|ref|NC_015612.1|	15395
Hepialidae	*Ahamus yunnanensis*	gi|393281654|ref|NC_018095.1|	15816
	Thitarodes renzhiensis	gi|393281640|ref|NC_018094.1|	16173
Hesperiidae	*Ctenoptilum vasava*	gi|372291669|ref|NC_016704.1|	15468
	Erynnis montanus	gi|511348601|ref|NC_021427|	15530
	Ochlodes venata	gi|391224062|ref|NC_018048.1|	15622
Lycaenidae	*Celastrina hersilia*	gi|391224076|ref|NC_018049.1|	15302
	Coreana raphaelis	gi|94490710|ref|NC_007976.1|	15314
	Cupido argiades	gi|470235912|ref|NC_020779.1|	15489
	Protantigius superans	gi|347600337|ref|NC_016016.1|	15248
	Spindasis takanonis	gi|347600365|ref|NC_016018.1|	15349
Lymantriidae	*Gynaephora menyuanensis*	gi|452849545|ref|NC_020342.1|	15770
Lyonetiidae	*Leucoptera malifoliella*	gi|403531566|ref|NC_018547.1|	15646
	Agrotis ipsilon	gi533207083refNC_022185.1	15377
	Agrotis segetum	gi556505731refNC_022689.1	15378
Noctuidae	*Ctenoplusia agnata*	gi|511348131|ref|NC_021410|	15261
	Helicoverpa armigera	gi|312233066|ref|NC_014668.1	15347
	Spodoptera litura	gi556505529refNC_022676.1	15388
	Ochrogaster lunifer	gi|195954010|ref|NC_011128.1|	15593
	Sesamia inferens	gi|339906831|ref|NC_015835.1|	15413
	Spodoptera exigua	gi|426406268|ref|NC_019622.1|	15365
Notodontidae	*Phalera flavescens*	gi|351000167|ref|NC_016067.1|	15659
Nymphalidae	*Acraea issoria*	gi|280978083|ref|NC_013604.1|	15245
	Apatura ilia	gi|351000139|ref|NC_016062.1|	15242
	Apatura metis	gi|333236263|ref|NC_015537.1|	15330
	Argynnis hyperbius	gi|345894229|ref|NC_015988.1|	15156
	Athyma sulpitia	gi|385099606|ref|NC_017744.1|	15268
	Calinaga davidis	gi|331746893|ref|NC_015480.1|	15267
	Danaus plexippus	gi|511348911|ref|NC_021452|	15314
	Euploea mulciber	gi|372292005|ref|NC_016720.1|	15166
	Fabriciana nerippe	gi|359421964|ref|NC_016419.1|	15140
	Heliconius melpomene	gi|429217734|ref|NC_019812|	15328
	Hipparchia autonoe	gi|308745928|ref|NC_014587.1|	15489
	Issoria lathonia	gi|391223809|ref|NC_018030.1|	15172
	Junonia orithya	gi556505845refNC_022697.1	15214
	Kallima inachus	gi|357018090|ref|NC_016196.1|	15183
	Libythea celtis	gi|372292061|ref|NC_016724.1|	15164
	Melanitis leda	gi|511347533|ref|NC_021370|	15122

		Melitaea cinxia	gi|391223795|ref|NC_018029.1|	15170
		Sasakia charonda	gi|297572345|ref|NC_014223.1|	15236
		Sasakia funebris	gi533206253refNC_022134.1	15233
		Timelaea maculata	gi|482660337|ref|NC_021090.1|	15178
	Papilionidae	*Luehdorfia chinensis*	gi|205277633|gb|EU622524	13860
		Papilio bianor	gi|391223948|ref|NC_018040.1|	15340
		Papilio dardanus	gi|402535439|gb|JX313686	13205
		Papilio maackii	gi|511348145|ref|NC_021411|	15357
		Papilio machaon	gi|391224048|ref|NC_018047.1|	15185
		Papilio maraho	gi|295065636|ref|NC_014055.1|	16094
		Papilio xuthus	gi|148373669|gb|EF621724	13964
		Parnassius bremeri	gi|295065608|ref|NC_014053.1|	15389
		Teinopalpus aureus	gi|302632610|ref|NC_014398.1|	15242
	Pieridae	*Aporia crataegi*	gi|400201563|ref|NC_018346.1|	15140
		Catopsilia pomona	gi556505702refNC_022687.1	15142
		Delias hyparete	gi|456351426|ref|NC_020428.1|	15186
		Eurema hecabe	gi556505673refNC_022685.1	15160
		Hebomoia glaucippe	gi|482651296|ref|NC_021123.1|	15701
		Pieris melete	gi|177807247|ref|NC_010568.1|	15140
		Pieris rapae	gi|342240289|ref|NC_015895.1|	15157
	Plutellidae	*Plutella xylostella*	gi|334849958|gb|JF9118	16179
	Pyralidae	*Corcyra cephalonica*	gi|375267107|ref|NC_016866.1|	15273
		Ephestia kuehniella	gi546143949refNC_022476.1	15295
		Ostrinia furnacalis	gi|18314304|ref|NC_003368.1|	14536
	Riodinidae	*Abisara fylloides*	gi|525340890|ref|NC_021746|	15301
	Saturniidae	*Actias selene*	gi|394831470|ref|NC_018133.1|	15236
		Antheraea pernyi	gi|162279939|ref|NC_004622.2|	15566
		Antheraea yamamai	gi|238694149|ref|NC_012739.1|	15338
		Attacus atlas	gi|525341170|ref|NC_021770|	15282
		Eriogyna pyretorum	gi|238563960|ref|NC_012727.1|	15327
		Samia cynthia ricini	gi|386800482|ref|NC_017869.1|	15384
		Saturnia boisduvalii	gi|184202694|ref|NC_010613.1|	15360
	Sphingidae	*Manduca sexta*	gi|165932395|ref|NC_010266.1|	15516
		Sphinx morio	gi|470235926|ref|NC_020780.1|	15299
	Tortricidae	*Acleris fimbriana*	gi|408772054|ref|NC_018754.1|	15933
		Adoxophyes honmai	gi|108793378|ref|NC_008141.1|	15680
		Adoxophyes orana	gi|511347949|ref|NC_021396|	15343
		Choristoneura longicellana	gi|435855628|ref|NC_019996.1|	15759
		Cydia pomonella	gi|443329593|ref|NC_020003|	15253
		Grapholita molesta	gi|315270918|ref|NC_014806.1|	15717
		Rhyacionia leptotubula	gi|426406226|ref|NC_019619.1|	15877
		Spilonota lechriaspis	gi|299829144|ref|NC_014294.1|	15368
Mantodea	Mantidae	*Tamolanica tamolana*	gi|84488776|ref|NC_007702.1|	16055
Mecoptera	Bittacidae	*Bittacus pilicornis*	gi|322830690|ref|NC_015118.1|	15842

	Boreidae	*Boreus elegans*	gi|322830704|ref|NC_015119.1|	16803
	Nannochoristidae	*Microchorista philpotti*	gi|321401409|gb|HQ696	19092
	Panorpidae	*Neopanorpa pulchra*	gi|256985280|ref|NC_013180.1|	15531
Megaloptera	Corydalidae	*Corydalus cornutus*	gi|205351318|ref|NC_011276.1|	15687
		Neochauliodes punctatolosus	gi|408772222|ref|NC_018772.1|	15734
		Protohermes concolorus	gi|211998787|ref|NC_011524.1|	15851
	Sialidae	*Sialis hamata*	gi|258649614|ref|NC_013256.1|	15608
Neuroptera	Ascalaphidae	*Ascalohybris subjacens*	gi|511348615|ref|NC_021428|	15873
		Ascaloptynx appendiculatus	gi|205351332|ref|NC_011277.1|	15877
		Libelloides macaronius	gi|334701684|ref|NC_015609.1|	15890
	Chrysopidae	*Apochrysa matsumurae*	gi|322422620|ref|NC_015095.1|	16214
		Chrysopa pallens	gi|426406212|ref|NC_019618.1|	16723
		Chrysoperla nipponensis	gi|322422592|ref|NC_015093.1|	16057
	Mantispidae	*Ditaxis biseriata*	gi|258649628|ref|NC_013257.1|	16416
	Osmylidae	*Thyridosmylus langii*	gi|511348201|ref|NC_021415|	16221
	Polystoechotidae	*Polystoechotes punctatus*	gi|205351290|ref|NC_011278.1|	16036
Odonata	Coenagrionidae	*Ischnura pumilio*	gi|517502124|ref|NC_021617|	15250
	Corduliidae	*Cordulia aenea*	gi|443298302|gb|JX963627	14488
	Euphaeidae	*Euphaea formosa*	gi|306960177|ref|NC_014493.1|	15700
	Gomphidae	*Davidius lunatus*	gi|229317937|ref|NC_012644.1|	15913
	Pseudolestidae	*Pseudolestes mirabilis*	gi|470229443|ref|NC_020636.1|	15122
Orthoptera	Acrididae	*Acrida cinerea*	gi|317097421|ref|NC_014887.1|	15599
		Acrida willemsei	gi|207268051|ref|NC_011303.1|	15601
		Arcyptera coreana	gi|288900664|ref|NC_013805.1|	15783
		Calliptamus italicus	gi|207269079|ref|NC_011305.1|	15675
		Chondracris rosea	gi|435855542|ref|NC_019993.1|	15646
		Chorthippus chinensis	gi|194871823|ref|NC_011095.1|	15599
		Euchorthippus fusigeniculatus	gi|304322824|ref|NC_014449.1|	15772
		Gastrimargus marmoratus	gi|195661170|ref|NC_011114.1|	15924
		Gomphocerippus rufus	gi|301353420|ref|NC_014349.1|	15598
		Gomphocerus licenti	gi|288904176|ref|NC_013847.1|	15597
		Gomphocerus sibiricus	gi|482651031|ref|NC_021103.1|	15590
		Gomphocerus s.tibetanus	gi|331746864|ref|NC_015478.1|	15571
		Lentula callani	gi|470235842|ref|NC_020774.1|	15944
		Locusta migratoria	gi|5835247|ref|NC_001712.1|	15722
		Locusta migratoria manilensis	gi|317097457|ref|NC_014891.1|	15895
		Locusta migratoria migratoria	gi|195933665|ref|NC_011119.1|	16053
		Locusta migratoria tibetensis	gi|334362226|ref|NC_015624.1|	15568
		Ognevia longipennis	gi|283098215|ref|NC_013701.1|	15621
		Phlaeoba albonema	gi|219524285|ref|NC_011827.1|	15657
		Prumna arctica	gi|288903464|ref|NC_013835.1|	15628
		Schistocerca gregaria	gi|258649420|ref|NC_013240.1|	15625
		Sclerophasma paresisense	gi|84488734|ref|NC_007701.1|	15500
		Shirakiacris shirakii	gi|517501955|ref|NC_021610|	15649

		Traulia szetschuanensis	gi|288903338|ref|NC_013826.1|	15768
		Xenocatantops brachycerus	gi|517501931|ref|NC_021609|	15605
	Caelifera	*Oedaleus decorus asiaticus*	gi|195661156|ref|NC_011115.1|	16259
	Conocephalidae	*Ruspolia dubia*	gi|157786563|ref|NC_009876.1|	14971
	Episactidae	*Pielomastax zhengi*	gi|357017937|ref|NC_016182.1|	15602
	Erianthinae	*Erianthus versicolor*	gi|435857150|ref|NC_020045.1|	15397
	Gryllinae	*Teleogryllus emma*	gi|219524229|ref|NC_011823.1|	15660
	Gryllotalpidae	*Gryllotalpa pluvialis*	gi|207269746|ref|NC_011302.1|	15525
		Gryllotalpa orientalis	gi|58045502|ref|NC_006678.1|	15521
	Lithidiidae	*Lithidiopsis carinatus*	gi|470235856|ref|NC_020775.1|	15652
	Myrmecophilidae	*Myrmecophilus manni*	gi|207268065|ref|NC_011301.1|	15323
	Oedipodidae	*Ceracris kiangsu*	gi|435855587|ref|NC_019994.1|	15665
	Ommexechidae	*Ommexecha virens*	gi|470235898|ref|NC_020778.1|	15536
	Oxyinae	*Oxya chinensis*	gi|164420921|ref|NC_010219.1|	15443
	Pamphagidae	*Asiotmethis zacharjini*	gi|452849276|ref|NC_020328.1|	15660
		Filchnerella helanshanensis	gi|452849290|ref|NC_020329.1|	15657
		Pseudotmethis rubimarginis	gi|452849304|ref|NC_020330.1|	15661
		Thrinchus schrenkii	gi|309259966|ref|NC_014610.1|	15672
	Pneumoridae	*Physemacris variolosa*	gi|306960149|ref|NC_014491.1|	17004
	Prophalangopsidae	*Tarragoilus diuturnus*	gi|511347963|ref|NC_021397|	16144
	Pyrgacrididae	*Pyrgacris descampsi*	gi|470235870|ref|NC_020776.1|	15618
	Pyrgomorphidae	*Atractomorpha sinensis*	gi|219524243|ref|NC_011824.1|	15558
		Mekongiana xiangchengensis	gi|304322838|ref|NC_014450.1|	15567
		Mekongiella xizangensis	gi|304322852|ref|NC_014451.1|	15885
	Rhaphidophoridae	*Troglophilus neglectus*	gi|207270760|ref|NC_011306.1|	15810
	Romaleidae	*Xyleus modestus*	gi|306960135|ref|NC_014490.1|	15723
	Tanaoceridae	*Tanaocerus koebelei*	gi|470235884|ref|NC_020777.1|	15515
	Tetrigidae	*Alulatettix yunnanensis*	gi|403531433|ref|NC_018542.1|	15104
		Tetrix japonica	gi|403531471|ref|NC_018543.1|	15128
	Tettigoniidae	*Anabrus simplex*	gi|159524429|ref|NC_009967.1|	15766
		Conocephalus maculates	gi|372291589|ref|NC_016696.1|	15898
		Deracantha onos	gi|219524090|ref|NC_011813.1|	15650
		Elimaea cheni	gi|299829075|ref|NC_014289.1|	15831
		Gampsocleis gratiosa	gi|197935811|ref|NC_011200.1|	15929
		Mecopoda elongata	gi|511347753|ref|NC_021380|	15284
		Mecopoda niponensis	gi|511347739|ref|NC_021379|	15364
		Sinochlora longifissa	gi|511348402|ref|NC_021424|	18133
		Sinochlora retrolateralis	gi|474452578|gb|KC467056	17209
		Xizicus fascipes	gi|408772152|ref|NC_018765.1|	16166
	Tridactylidae	*Ellipes minuta*	gi|306960090|ref|NC_014488.1|	15451
	Tristiridae	*Tristira magellanica*	gi|470235828|ref|NC_020773.1|	16494
Phasmatodea	Bacillidae	*Bacillus atticus*	gi|291062083|gb|GU001955	14141
		Bacillus rossius	gi|261840594|gb|GU001956	14152
	Diapheromeridae	*Micadina phluctainoides*	gi|312233136|ref|NC_014673.1|	16867

	Heteropterygidae	*Heteropteryx dilatata*	gi|312233193|ref|NC_014680.1|	16618
	Phasmatidae	*Entoria okinawaensis*	gi|313247873|ref|NC_014694.1|	16910
		Extatosoma tiaratum	gi|385137030|ref|NC_017748.1|	16537
		Megacrania alpheus adan	gi|312233307|ref|NC_014688.1|	17124
		Phobaeticus serratipes	gi|312233164|ref|NC_014678.1|	16182
		Phraortes illepidus	gi|313199763|ref|NC_014695.1|	16456
		Phraortes sp.	gi|312233493|ref|NC_014705.1|	16867
		Ramulus hainanense	gi|256985350|ref|NC_013185.1|	15590
		Ramulus irregulariterdentatus	gi|312233451|ref|NC_014702.1|	16633
Phasmida	Timematidae	*Timema californicum*	gi|82541839|gb|DQ241799	14387
Phthiraptera	Boopidae	*Heterodoxus macropus*	gi|12383036|ref|NC_002651.1|	14670
	Philopteridae	*Anaticola crassicornis*	gi|345894324|ref|NC_015998.1|	8118
		Bothriometopus macrocnemis	gi|160425216|ref|NC_009983.1|	15564
		Campanulotes bidentatus	gi|89257204|ref|NC_007884.1|	14804
		Coloceras sp.	gi|345894310|ref|NC_015997.1|	14869
		Ibidoecus bisignatus	gi|345894330|ref|NC_015999.1|	14908
Plecoptera	Pteronarcyidae	*Pteronarcys princeps*	gi|51101192|ref|NC_006133.1|	16004
Psocoptera	Lepidopsocidae	*Lepidopsocid* sp.	gi|31324905|ref|NC_004816.1|	16924
	Psocidae	*Longivalvus hyalospilus*	gi|426262340|gb|JQ910986	14442
		Psococerastis albimaculata	gi|511347991|ref|NC_021400|	15589
Raphidioptera	Raphidiidae	*Mongoloraphidia harmandi*	gi|258649574|ref|NC_013251.1|	16006
Siphonaptera	Ceratophyllidae	*Jellisonia amadoi*	gi556506033refNC_022710.1	17031
Strepsiptera	Mengenillidae	*Mengenilla australiensis*	gi|281335169|gb|GU188852	13421
		Mengenilla moldrzyki	gi|403531501|ref|NC_018545.1|	15363
	Stylopidae	*Xenos vesparum*	gi|86142538|gb|DQ36	14519
Thysanoptera	Thripidae	*Frankliniella intonsa*	gi|511347725|ref|NC_021378|	15215
		Frankliniella occidentalis	gi|400201896|ref|NC_018370.1|	14889
		Thrips imaginis	gi|25057389|ref|NC_004371.1|	15407
Thysanura	Lepidotrichidae	*Tricholepidion gertschi*	gi|42632243|ref|NC_005437|	15267
	Lepismatidae	*Thermobia domestica*	gi|50812159|ref|NC_006080.1|	15152
Zygentoma	Ateluridae	*Atelura formicaria*	gi|197935825|ref|NC_011197.1|	15205
	Lepidotrichidae	*Tricholepidion gertschi*	gi|42632243|ref|NC_005437.1|	15267

Table B. Mitochondrial baits used for the species assignment of the contigs/scaffolds assembled for mitogenome elucidation, according to methodology in Paula *et al*. (2015).

Target species	Gene fragments	bp	GenBank code
Aphis gossypii	COI	1291	gi|428230607|gb|JQ860257.1|
	CytB	724	gi|31298385|gb|HQ528289.1|
	tRNA-Leu, COII, tRNA-Lys	1,464	gi|378407886|gb|JQ067122.1|
	tRNA-Asn to ND5	2,029	gi|551682696|gb|KF636759.1|
	12S rRNA, tRNA-Val, 16S rRNA	1,600	gi|169821740|gb|EU358872.1|
Chrysoperla carnea	COI	876	KJ849286
	16S	474	gi|58271788|gb|AY743763.1|
	COII	685	gi|380035151|dbj|AB671878.1|
	ND5	1,719	gi|380035479|dbj|AB672042.1|
	ND2	1,000	gi|38003515|dbj|AB671960.1|
	COIII	667	gi|48728241|gb|AY620064.1|
Chrysoperla externa	COI	821	gi|89520560|gb|DQ414490.1|
	CytB	470	gi|58271970|gb|AY743862.1|
	16S	556	gi|89146869|gb|DQ399271.1|
	COII	578	gi|58271884|gb|AY743819.1|
Coleomegilla maculata	COI	1132	gi|47680450|gb|AY615732.1|
	CytB		KJ849292
	16S	325	gi|283463879|gb|GU073845.1|
	12S	289	gi|283463824|gb|GU073790.1|
Cycloneda munda	COI	876	KJ849284
	CytB	410	KJ849290
Cycloneda sanguinea	COI	878	KJ849287
Doru luteipes	COI	879	KJ849285
	CytB	344	KJ849291
Euschistus variolarius	COI	877	KJ849288
Harmonia axyridis	COI	1,188	gi|409979052|gb|JQ715429.1|
	CytB	557	gi|302123994|gb|HM122754.1|
	16S	537	gi|222431111|gb|FJ601204.1|
	12S	394	gi|326417566|gb|HQ644021.1|
	COII	688	gi|159906287|gb|EU106638.1|
Helicoverpa zea	COI	1272	gi|310617293|gb|HQ177284.1|
	CytB	1015	gi|310617949|gb|HQ177612|
	16S	418	gi|310618887|gb|HQ178247.1|
	12S	290	gi|310618658|gb|HQ178018.1|
	COII	700	gi|315229651|gb|HQ677777.1|
	COII, tRNA-Leu, COIII	605	gi|212292258|gb|EU918929.1|
Hippodamia convergens	COI	820	gi|161318286|gb|EU164681.1|
	CytB	496	KJ849293
	COII	641	gi|315229747|gb|HQ677825.1|
	16S	350	gi|161318246|gb|EU164588.1|
Hippodamia tredecempunctata	COI	876	KJ849289
Myzus persicae	COI	1563	gi|297501415|gb|AB506741.1|
	CytB	871	gi|312983853|gb|HQ528298.1|
Orius insidiosus	COI	874	KJ849283
	CytB	391	gi|298578880|gb|GQ384368.1|

	16S	400	gi|298578785|gb|GQ258390.1|
Rhopalosiphon maidis	COI	1291	gi|428230623|gb|JQ860265.1|
	CytB	1006	gi|22208806|emb|AJ315893.1|
	12S, tRNA-Val, 16S	1590	gi|169821762|gb|EU358894.1|
	tRNA-Leu, COII	702	gi|29648626|gb|AY219738.1|
Spodoptera frugiperda	COI	1263	gi|310617429|gb|HQ177352.1|
	CytB	1015	gi|310618073|gb|HQ177674.1|
Thrips tabaci	COI	810	gi|139001405|gb|AB26442|
	16S	299	gi|42564970|bg|AY523655.1|
	COIII	216	gi|169238893|emb|AM941963.1|

Table C. Mitochondrial DNA elucidated for the target species, according to methodology in Timmermans *et al.* 2010, Crampton-Platt *et al.* (2015) and in Paula *et al.* (2015).

**Species**		**GenBank code**	**Size (bp)**
**Predators**			
Coleoptera: Coccinellidae			
	Coleomegilla maculata	KJ778881	17,517
	Cycloneda munda	KJ778882	14,293
	Cycloneda sanguinea	KJ778883	15,137
	Harmonia axyridis	KJ778886	15,322
	Hippodamia convergens	KJ778888	12,618
	Hippodamia tredecimpunctata	KJ778889	17,281
Neuroptera: Chrysopidae	*Chrysoperla carnea*	KJ778879	10,856
	Chrysoperla externa	KJ778880	3,706
Dermaptera: Forficulidae	*Doru luteipes*	KJ778884	9,246
Hemiptera: Anthocoridae	*Orius insidosus*	KJ778890	14,472
**Prey**			
Hemiptera			
Aphididae	*Aphis gossypii*	-	baits (7,108)
	Myzus persicae	-	baits (1,307)
	Rhopalosiphum maidis	KJ778891	13,781
Pentatomidae	*Euschistus variolarius*	KJ778885	12,077
Lepidoptera: Noctuidae			
	Helicoverpa zea	KJ778887	15,338
	Spodoptera frugiperda	KJ778892	15,328
Thysanoptera: Thripidae	*Thrips tabaci*	-	1,984 and baits (1,795)

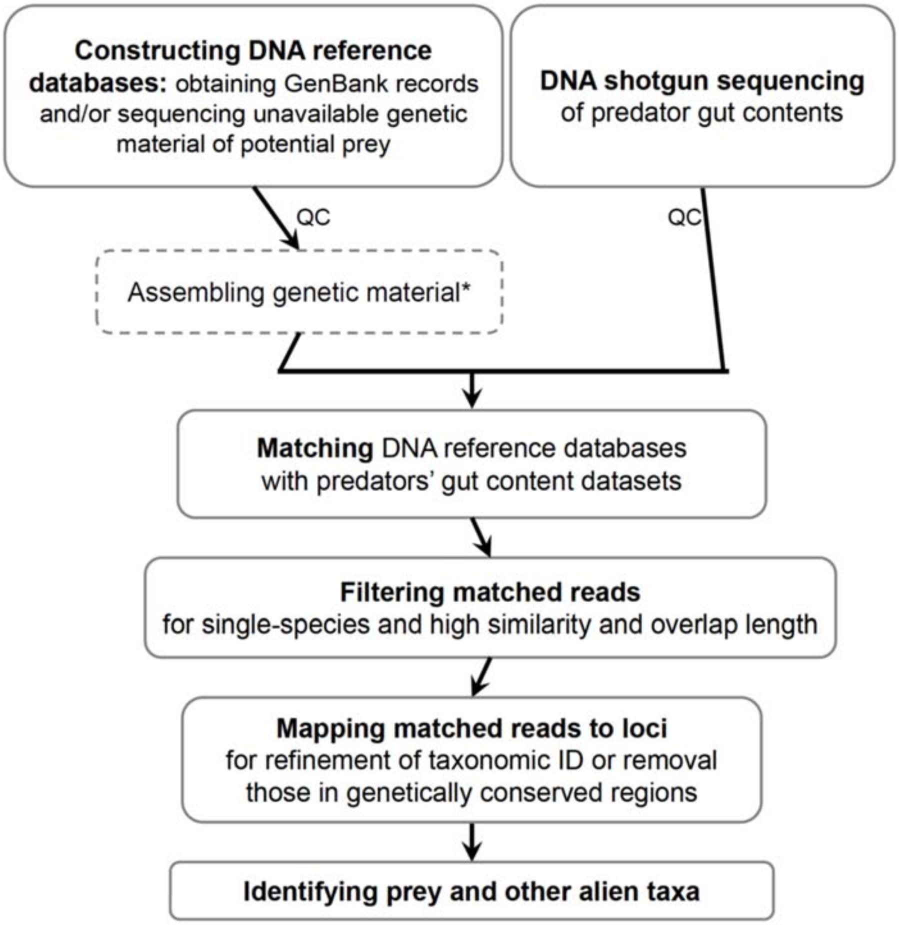

Fig. A. Pipeline for using DNA shotgun-sequencing to identify trophic interactions of generalist arthropod predators. To construct DNA reference database of an unavailable potential prey, either the mitogenome or the nuclear genome (its scaffolds) would be preferential, instead of just get some barcode genes. * The assembly step only would be necessary in the cases when the elucidation of a genome (mito or nuclear) is required.

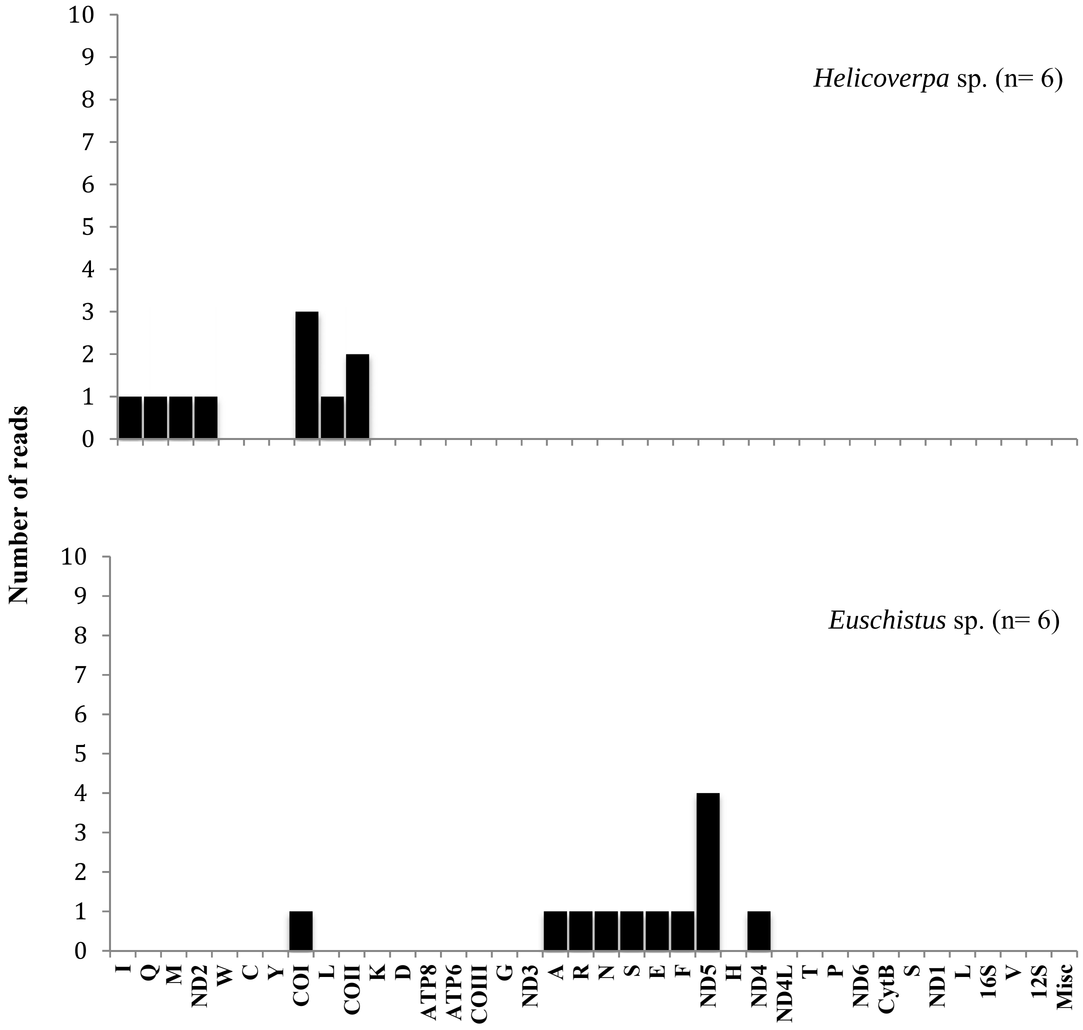
Number of reads
Helicoverpa sp. (n= 6)
Euschistus sp. (n= 6)
I Q M ND2 W C Y COI L COII K D ATP8 ATP6 COIII G ND3 A R N S E F ND5 H ND4 ND4L T P ND6 CytB S ND1 L 16S V 12S Misc

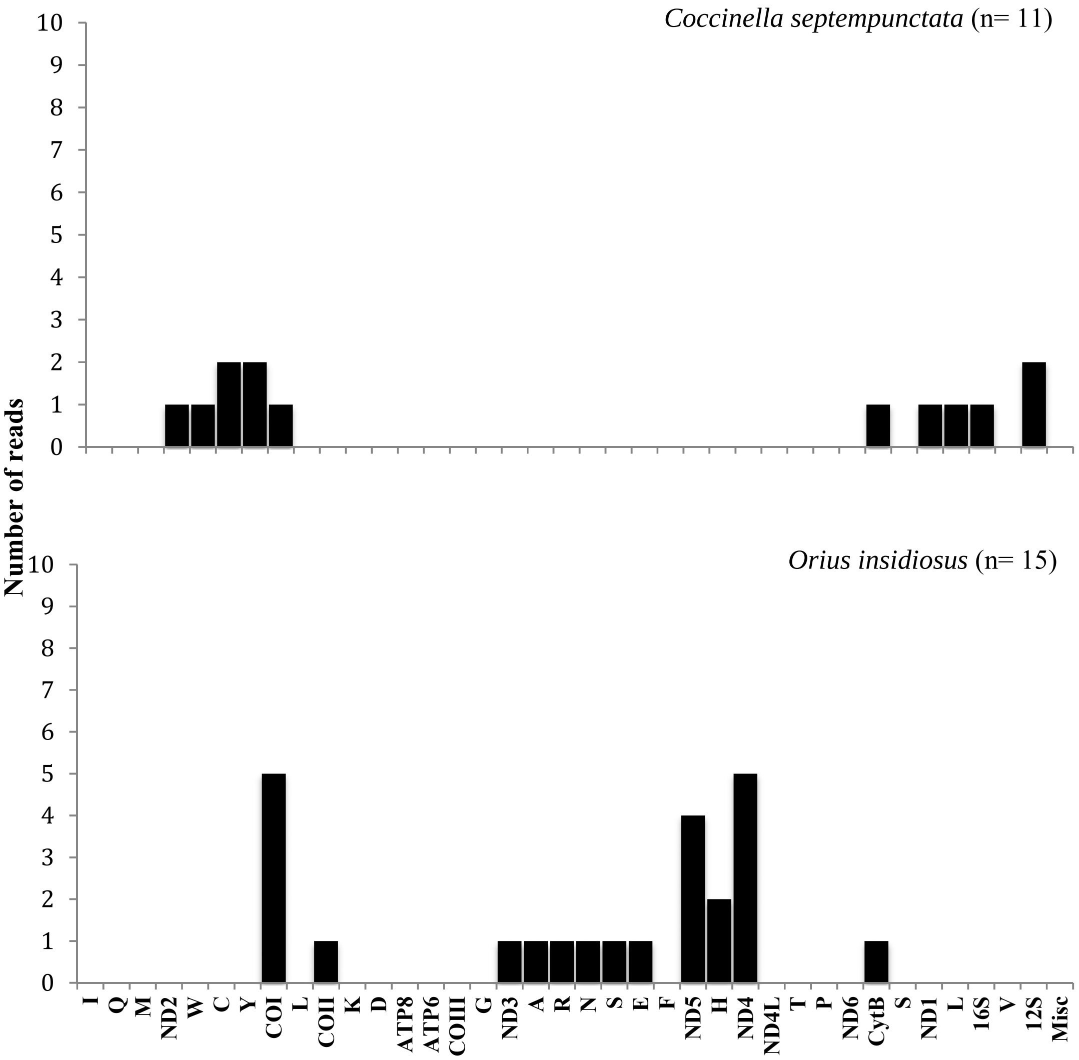
Coccinella septempunctata (n= 11)
Orius insidiosus (n= 15)
Number of reads
0
1
2
3
4
5
6
7
8
9
10
I
Q
M
ND2
W
C
Y
COI
L
COII
K
D
ATP8
ATP6
COIII
G
ND3
A
R
N
S
E
F
ND5
H
ND4
ND4L
T
P
ND6
CytB
S
ND1
L
16S
V
12S
Misc

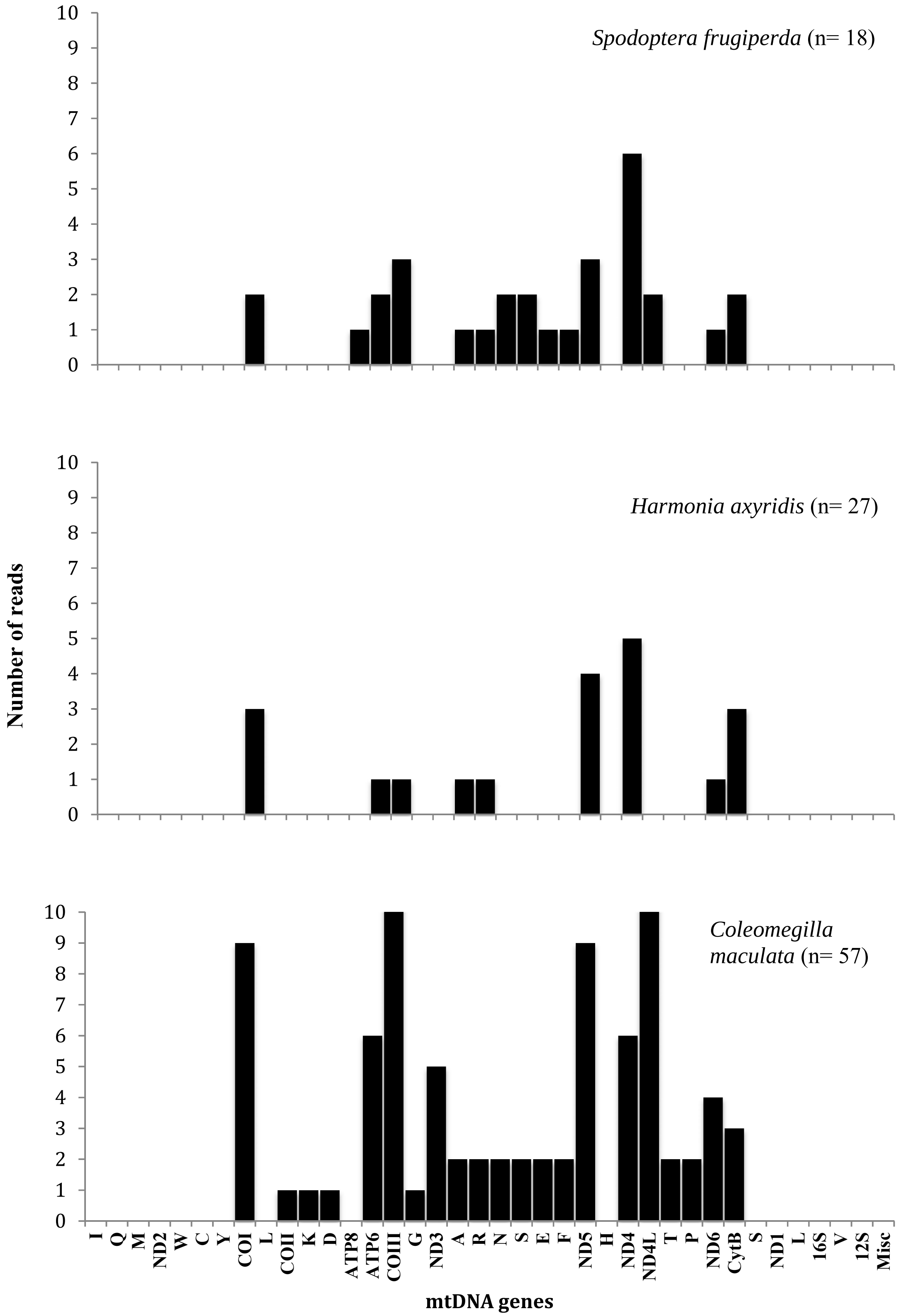

Fig. B. Mapping of reads to mitogenomes of prey found in *Hippodamia convergens*. The total number of genes in the figures does not always match with the number of reads detected (Table 2) because some reads mapped across more than one gene or in an intergenic region.

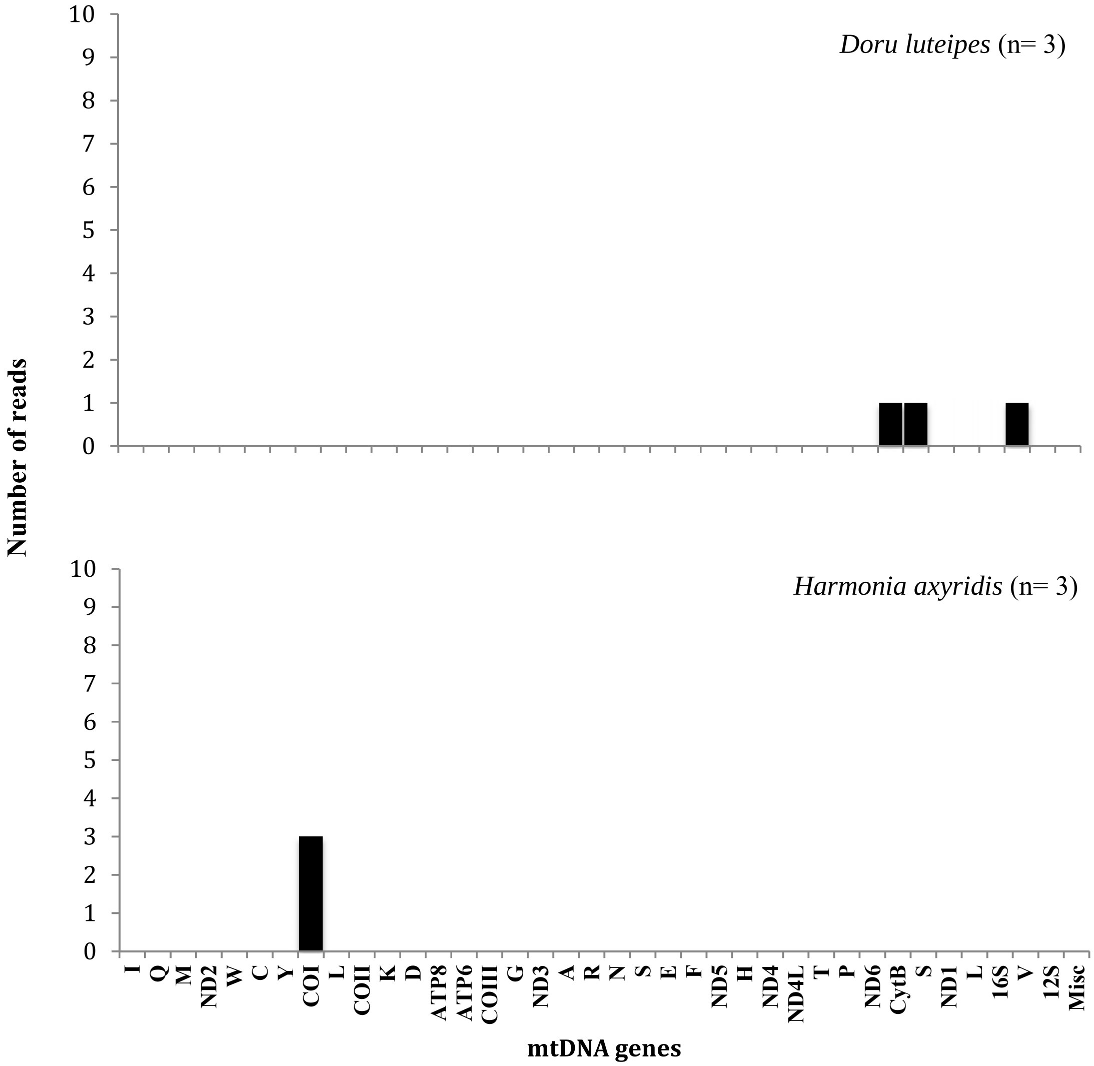

Fig. C. Mapping of reads to mitogenomes of prey found in *Cycloneda sanguinea*. The total number of genes in the figures does not always match with the number of reads detected (Table 2) because some reads mapped across more than one gene or in an intergenic region.

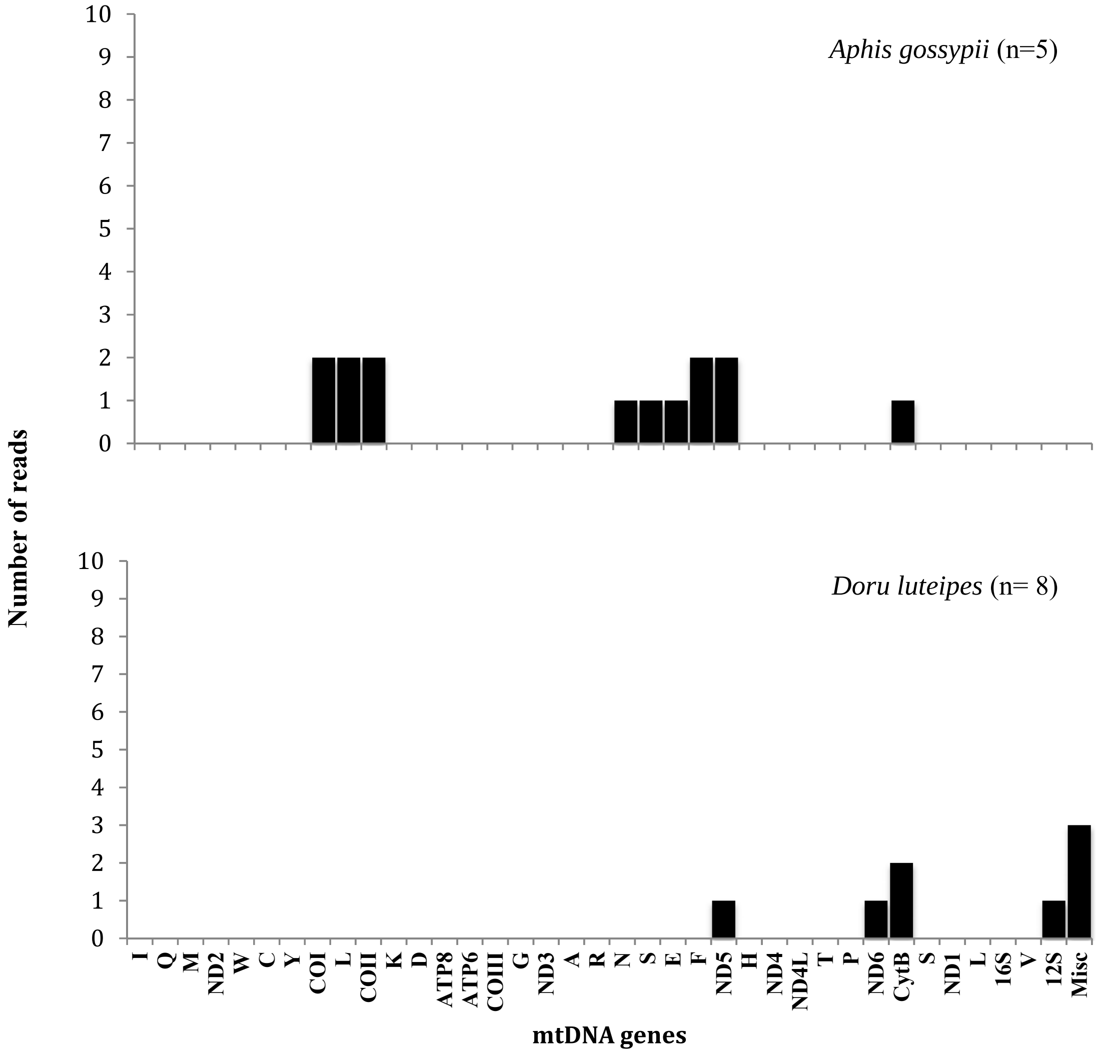

Fig. D. Mapping of reads to mitogenomes of prey found in *Harmonia axyridis*.

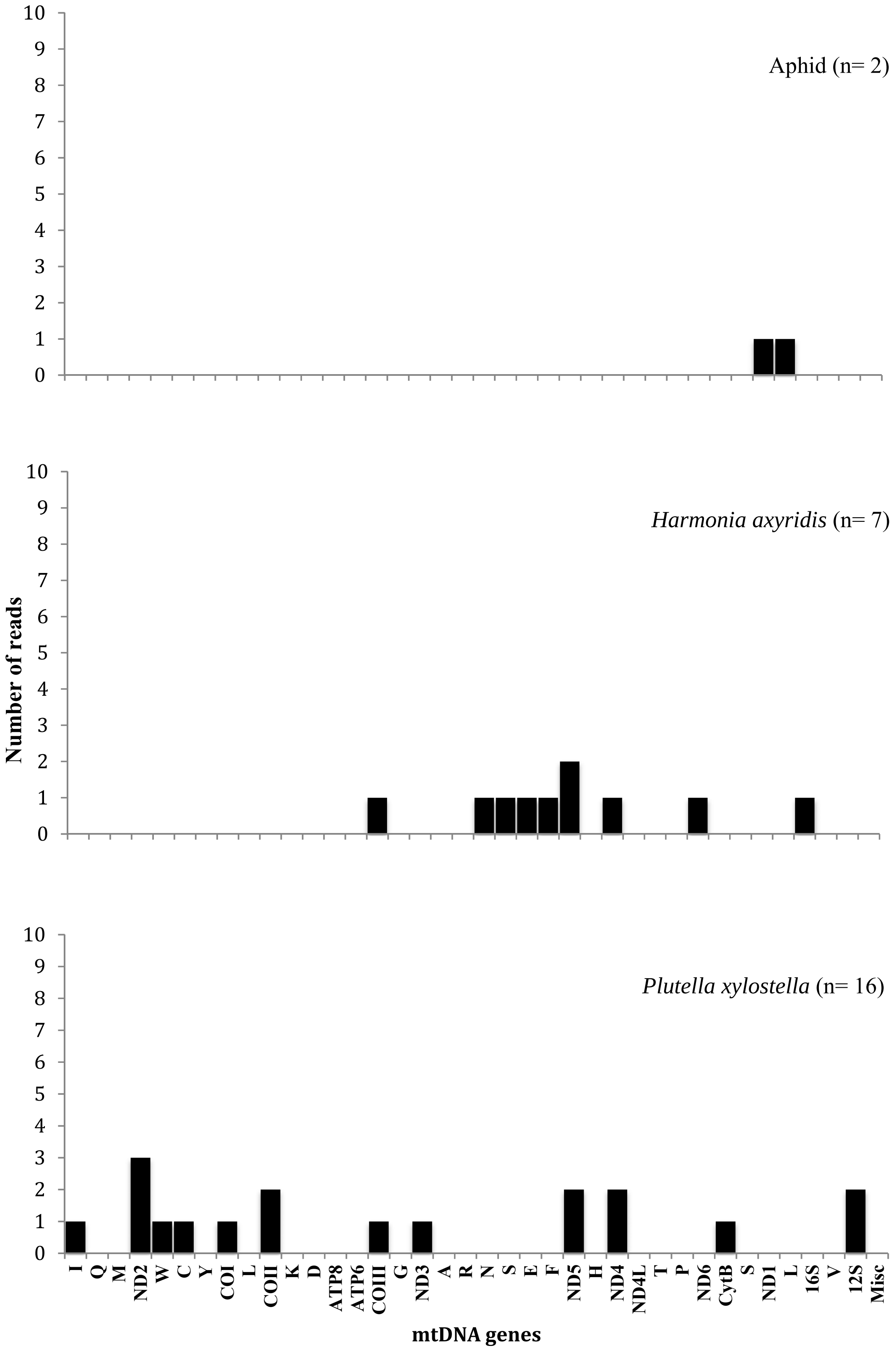

Fig. E. Mapping of reads to mitogenomes of prey found in *Doru luteipes*.
